# Integration of proprioceptive and visual feedback during online control of reaching

**DOI:** 10.1152/jn.00639.2020

**Published:** 2021-12-15

**Authors:** Shoko Kasuga, Frédéric Crevecoeur, Kevin P. Cross, Parsa Balalaie, Stephen H. Scott

**Affiliations:** ^1^Centre for Neuroscience Studies, Queen’s University, Kingston, Ontario, Canada; ^2^Institute of Communication Technologies, Electronics and Applied Mathematics, Louvain-la-Neuve, Belgium; ^3^Institute of Neuroscience, Université Catholique de Louvain, Louvain-la-Neuve, Belgium; ^4^Department of Biomedical and Molecular Sciences, Queen’s University, Kingston, Ontario, Canada; ^5^Department of Medicine, Queen’s University, Kingston, Ontario, Canada

**Keywords:** feedback control, goal-directed movements, human, multisensory integration, state estimation

## Abstract

Visual and proprioceptive feedback both contribute to perceptual decisions, but it remains unknown how these feedback signals are integrated together or consider factors such as delays and variance during online control. We investigated this question by having participants reach to a target with randomly applied mechanical and/or visual disturbances. We observed that the presence of visual feedback during a mechanical disturbance did not increase the size of the muscle response significantly but did decrease variance, consistent with a dynamic Bayesian integration model. In a control experiment, we verified that vision had a potent influence when mechanical and visual disturbances were both present but opposite in sign. These results highlight a complex process for multisensory integration, where visual feedback has a relatively modest influence when the limb is mechanically disturbed, but a substantial influence when visual feedback becomes misaligned with the limb.

**NEW & NOTEWORTHY** Visual feedback is more accurate, but proprioceptive feedback is faster. How should you integrate these sources of feedback to guide limb movement? As predicted by dynamic Bayesian models, the size of the muscle response to a mechanical disturbance was essentially the same whether visual feedback was present or not. Only under artificial conditions, such as when shifting the position of a cursor representing hand position, can one observe a muscle response from visual feedback.

## Graphical abstract

**Figure:**
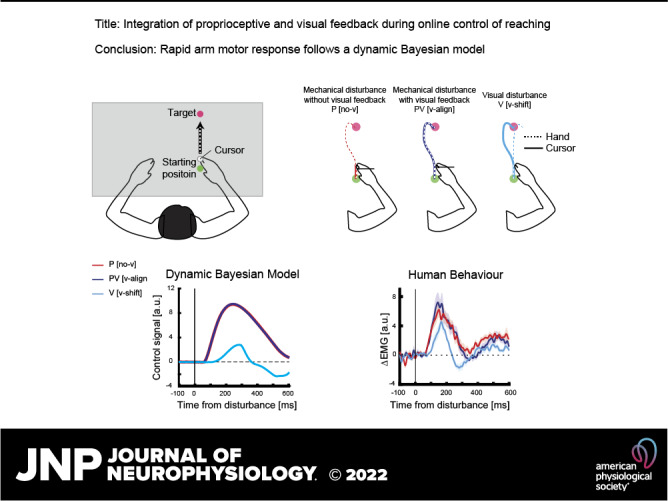

## INTRODUCTION

Many studies highlight the importance of different sensory modalities for generating corrections during motor actions (1–3). Although it is clear that visual and proprioceptive feedback can generate rapid motor responses to counter errors or disturbances, most research has focused on exploring how each sensory modality on its own influences motor output. These studies highlight that mechanical disturbances of the arm elicit a spinal stretch reflex in as little as 25 ms that predominantly considers disturbance size, and a second response beginning at 60 ms that includes transcortical feedback and considers a broad range of contexts such as target redundancy and the presence of obstacles (4–8). Similarly, visual shifts elicit two different motor responses: one motor response starting at 90 ms that considers the shift size, task relevance of the disturbances (i.e., statistical properties of the environment), and the distance to the target at the point a disturbance was applied (9–14). This is followed by a second response starting at 130 ms that considers more complex factors such as the presence of obstacles (10). These may reflect the presence of different feedback pathways including both subcortical and cortical contributions (10, 15–17).

Although most studies have focused on either visual or proprioceptive feedback for online control, we know very little on how these sensory modalities interact when correcting for motor errors. Two critical aspects add complexity to this question. First, proprioceptive feedback is always present as the receptors reside in the muscles used to move the limb, whereas visual information is conditional, as we are able to reach in the dark or with our eyes closed. Second, as shown in the literature on unimodal motor corrections, there is asynchrony in the times when visual and proprioceptive sources of feedback about the same disturbance become available. How does the presence of this context-dependent and delayed visual feedback influence motor corrections to mechanical disturbances?

Previously, we developed a dynamic Bayesian model of multisensory integration that accounts for asynchronous delays between sensory signals (18). A key element of dynamic Bayesian integration is the incorporation of a forward model about the plant that could be used to infer the current state of the limb from delayed sensory feedback from multiple sources. A prediction of dynamic Bayesian integration is that corrections to mechanical loads are largely unaffected by whether visual feedback of the hand is present or absent with only a reduction in the variance of the hand’s end point when vision was provided. Furthermore, experiments in humans demonstrated a similar pattern of integration with visual feedback only affecting the end point variance following a mechanical load.

However, one limitation of our previous study was that we could not address when dynamic Bayesian integration emerged in the motor system. Indeed, recent theories have speculated that the motor system exhibits a two-stage process for multisensory integration (19). An initial stage of sensory processing where each sensory modality is kept separate and updates their own intramodal state estimate as a way to quickly correct for errors. This is followed by a later stage of sensory processing that combines sensory modalities into a multimodal state estimate and could reflect dynamic Bayesian integration. The key prediction from the two-stage model is that the earliest corrective response for a combined visual and proprioceptive disturbance (bimodal disturbance) should reflect a linear sum of the disturbances applied when each sensory modality is disturbed separately (unimodal disturbance).

Here, we investigated the rules by which early corrective responses combine proprioceptive and visual feedback of the limb. As mentioned, the two-stage hypothesis assumes that the earliest corrective response to a bimodal disturbance will reflect a linear sum of the corrective responses for the equivalent unimodal disturbances. In contrast, we demonstrate that an emergent property of dynamic Bayesian integration is that a correction to a bimodal disturbance will appear as a subadditive sum of the corrections to the unimodal disturbances. We tested these models in human participants performing goal-directed reaches while unexpected mechanical loads were applied to the limb. We observed that when visual and mechanical disturbances were similar in size kinematically, the motor response for mechanical disturbances was always bigger than for visual disturbances. Critically, mechanical disturbances with visual feedback were not a simple addition of the corrective responses for the unimodal disturbances and instead were subadditive consistent with dynamic Bayesian integration. However, this did not mean visual feedback was unimportant, as confirmed in our second experiment highlighting that vision was potent when conflicts between proprioceptive and visual feedback were introduced. In all, we clearly establish that visual feedback has minimal impact when proprioceptive and visual feedback are congruent but can have a strong contribution when feedback modalities convey incongruent information.

## MATERIALS AND METHODS

### Participants

A total of 24 neurologically healthy individuals (8 females and 16 males, aged 18–38 yr, 20 right-hand dominant and 4 left-hand dominant) participated in one of the two experiments. *Experiments 1* and *2* included 13 and 12 participants, respectively. One participant completed both experiments. The interval between participation in separate experiments was more than 2 wk. Participants were financially compensated for their time. This study was conducted according to the Declaration of Helsinki. The experimental procedures were approved by the Queen’s University Research Ethics Board. Written informed consent was obtained from all participants before the experiments.

### Apparatus

**Figure 1.**
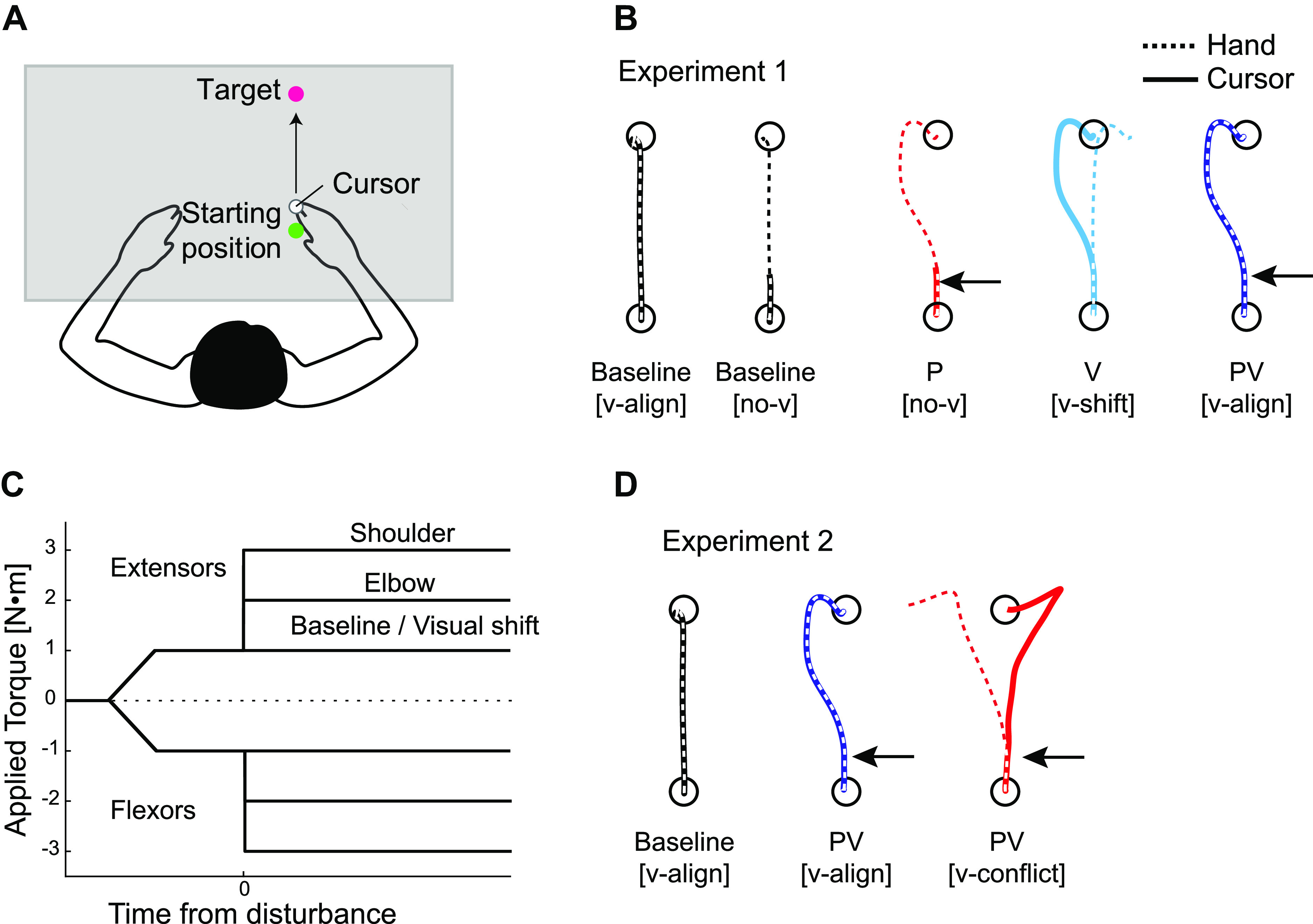
Experimental procedures. *A*: overhead view of the general experimental setup. The arms of participants were occluded by a screen under the display. Participants were asked to hit the target that was located 20 cm away from the start position by a cursor as quickly and straightly as possible. *B*: trial types in *experiment 1*. Dotted lines indicate hand trajectories and solid lines indicate cursor trajectories. Overlapped dotted and solid lines indicate that the hand and the cursor were aligned. Baseline, no disturbances applied; [no-v], no visual feedback provided; P, mechanical disturbance only; PV, mechanical and visual disturbance; V, visual disturbance only; [v-align], visual feedback present and aligned with hand; [v-shift], visual shift of cursor. *C*: time courses of applied torque. A background load of ±1 N·m was slowly introduced (ramp up, 500 ms) to prime muscle activities in either the elbow and shoulder flexors (negative signs) or extensors (positive signs), regardless of disturbance types (visual/mechanical). The background load was also applied in baseline trials. After a random holding period, a step torque was applied to both shoulder (additional 2 N·m) and elbow (additional 1 N·m) in trials with mechanical disturbance. *D*: trial types in *experiment 2* in the same format as in *B*. [v-conflict], Visual disturbance opposite direction to mechanical disturbance.

The experiment was performed using the Kinarm Exoskeleton Lab (Kinarm, Kingston ON, Canada; 20, 21). Participants sat in a chair and a Kinarm robot maintained the right arm in the horizontal plane. Torque motors attached to the linkages could apply mechanical loads to the elbow and/or shoulder joints. A virtual reality system displayed a white cursor aligned with the position of the right index fingertip (white circle of 10 mm diameter) and visual targets in the horizontal workspace (Fig. 1*A*). A barrier under the display prevented the participants from directly seeing their arms. The position and velocity of the hand was sampled at 1 kHz for offline analysis.

### General Task Procedure

Participants performed repetitive reaching movements starting from a position near the body (green circle, 20 mm diameter) toward a second spatial target 20 cm directly ahead (magenta circle, 20 mm diameter). The start target was located 5 cm toward the body from the point where the shoulder angle was ∼20° and the elbow angle ∼110°. If the participant was not able to reach this position, the start position was adjusted within 5 cm so that it became reachable.

Participants were instructed to move the cursor quickly from the start position to the target. Before each trial, the subject positioned the cursor at the start position for 3,000–3,750 ms. The target did not appear if the cursor left the start position during this period. Participants were asked to start the reach as soon as possible when the target appeared, although there was no explicit time constraint.

Participants were required to stop with the cursor inside the second target within 700 ms (unperturbed trials) or 1,100 ms (any perturbed trials) and remain at the target for at least 50 ms. To provide feedback to participants on whether they had met these criteria, we changed the color of the target after each trial. If they succeeded, the target turned green. If the movement was not completed within the time limit, the target disappeared and the word “slow” appeared instead.

After each trial, participants had to return the cursor/hand to the start position. They practiced ∼60 trials to become familiar with the task before the experiment.

### Experiment 1: Influence of Visual and/or Proprioceptive Feedback on Motor Corrections

This experiment examined how motor corrections generated by visual or proprioceptive errors are impacted when they are present on their own compared with when both modalities are present together. Experiments consisted of 400 trials in total and lasted for 1.5 h. There are four basic trial types (Fig. 1*B*). All trial types and conditions were randomly interleaved, and thus, participants were not able to anticipate the nature of the upcoming disturbance. Note that the background loads were applied for all trial types and were present during the entire trial (i.e., hold phase before the reach and during reach). The first trial type was baseline reaching in which there were no mechanical or visual disturbances applied during reaching (Fig. 1*B*, black lines). There were four conditions for these trials, which varied the background loads and the presence/absence of visual feedback of the hand. These trials provided baselines for different disturbance trial types. For one condition, a background load was applied that flexed the shoulder and elbow joints (1 N·m both joints; Fig. 1*C*). The cursor was present throughout the reach (v-align). In a different condition, the same reach was performed except that the cursor disappeared 3 cm past the start position and never appeared until the trial ended, thus requiring the participants to only use proprioceptive feedback to reach the goal (Baseline [no-v]). Participants also did not receive visual feedback of the cursor at the end point either. The remaining two conditions were the same as the previous two conditions except that the background load now extended the shoulder and elbow joints (1 N·m both joints). Different background loads were applied to observe both increases and decreases of muscle activities following the applied disturbance (22). These trial types consisted of 250 trials in total, 150 trials of conditions with cursor visual feedback and 100 trials without cursor visual feedback. Flexor and extensor background loads were applied with the same frequency.

The second trial type provided only proprioceptive feedback when mechanical disturbances were applied during reaching (Fig. 1*B*, P [no-v]). In this trial type, a mechanical load was applied when the hand was 3 cm past the start position and the cursor feedback was removed at the same time as when the load was applied. There were two mechanical disturbance conditions for these trials (Fig. 1*C*): combined flexor or combined extensor torques (elbow = 1 N·m and shoulder = 2 N·m). Flexor and extensor disturbances displaced the arm to the right and left, respectively. Flexor disturbances were coupled with flexor background loads, and extensor disturbances with extensor background loads. This trial type consisted of 50 trials in total, 25 trials for each condition.

The third trial type examined motor corrections when there was only a visual disturbance applied (Fig. 1*B*, V [v-shift]). To compare the muscle responses for visual versus mechanical disturbances, it was necessary to match the kinematics of the visual perturbation with the kinematics of the perturbation-related motion. Thus, we did not adopt the commonly used cursor jump procedure, but instead, we displaced cursor positions following trajectories that mimicked trajectories when the participant’s arm was mechanically perturbed. In this way, when participants moved their arm straight forward to the target the cursor shifted laterally as if the arm was pushed away, although there was no mechanical load applied. To make the trajectory (i.e., cursor position [*c*]) for visual disturbance trials, we estimated the amplitude (*a*), the abscissa (i.e., time) of the inflection point (*t_s_*), and slope (τ) of the sigmoid function (*Eq. 1*) using the position data of mechanical disturbance trials during a calibration session (see *Calibration session* at the end of this section) with a time epoch of 100 ms before disturbance to 250 ms after disturbance. The parameter *t* indicates the time from disturbance onset, and *h* indicates hand position. Each parameter was independently estimated for *x* and *y* axes.

(*1*) $$c\left(t\right)=h\left(t\right)+\frac{a}{1+e^{\frac{-\left(t+t_{s}\right)}{\text{τ}}}}.$$

There were two conditions for these trials, rightward and leftward cursor shifts, which corresponded to trajectories for the flexor and extensor mechanical disturbances, respectively. Since there was slightly different disturbance-related motion, we fitted parameters for flexor and extensor mechanical disturbances independently. Finally, either combined flexor or extensor background loads were again introduced with rightward or leftward cursor shift, respectively (same loads as in *experiment 1*). This trial type consisted of 50 trials in total, 25 trials for each condition.

The fourth trial type was similar to the second trial type where a mechanical disturbance was applied during reaching but now visual feedback of the hand was provided (Fig. 1*B*, PV [v-align]). Visual errors and proprioceptive errors were aligned. In this trial type, step torques were again applied to the right arm 3 cm past the start position. In addition, as with the second trial type, there were two conditions for these trials, combined flexor and extensor disturbances which were coupled with flexor and extensor background loads, respectively. This trial type consisted of 50 trials in total, 25 trials for each condition.

All trials types for *experiments 1* and *2* are summarized in a table (Supplemental Table S1; see https://github.com/KevinCross/DynamicBayesianModel).

#### Calibration session.

To determine the amplitude of the cursor shift individually, each participant completed a calibration session before the main experiment. The calibration session interleaved 40 unperturbed trials and 20 mechanical disturbance trials (10 flexion and 10 extension, same loads as in *experiment 1*), for a total of 60 trials.

### Experiment 2: Motor Correction When There is a Conflict between Proprioceptive and Visual Errors

*Experiment 2* examined how proprioceptive and visual feedback interact when they provide conflicting errors. Experiments consisted of 250 trials in total and lasted for ∼1 h. There were three basic trial types (Fig. 1*D*), which were randomly interleaved. The first trial type was baseline reaching in which there were no mechanical disturbances applied during reaching (Baseline [v-align]). We applied one of two background load conditions (combined flexor or combined extensor, same as *experiment 1*). Visual feedback of the cursor representing hand position was provided in all these trials. This trial type consisted of 150 trials in total, 75 trials for each condition.

The second trial type was exactly the same as the fourth trial type of *experiment 1*, which provided both visual and proprioceptive feedback when mechanical disturbances were applied during reaching (Fig. 1*D*, PV [v-align]). Visual errors and proprioceptive errors were aligned. This trial type consisted of 50 trials in total, 25 trials for each condition.

The third trial type provided both visual and proprioceptive feedback when mechanical disturbances were applied (same loads as in *experiment 1*), and visual disturbances were applied at the same time (Fig. 1*D*, PV [v-conflict]). Notably, in these trials visual errors and proprioceptive errors conflicted with each other. We applied mechanical disturbances to the arm while the cursor shifted from the hand position at the same time, so that the lateral error in proprioceptive and visual feedback occurred in the opposite directions. To realize this, the lateral position (i.e., *x*-axis) of the cursor was displaced from the lateral position of the hand following the sigmoid function described in *Eq. 2*.

(*2*) $$c_{x}\left(t\right)=h_{x}\left(t\right)+\frac{-2a_{x}}{1+e^{\frac{-\left(t+t_{s_{x}}\right)}{\text{τ}}}}.$$

The difference between visual disturbances of *experiments 1* and *2* is the amplitude and sign (i.e., direction) of the cursor shift, which appears in the coefficient of *a_x_*. In *experiment 2*, because the sign is reversed and the amplitude is doubled, the cursor (visual feedback) shifted in the opposite lateral direction to the hand (proprioceptive feedback). This manipulation enables us to dissociate motor correction to visual error and proprioceptive error by the direction of the movement. For vertical position (i.e., *y*-axis), we used the same function as *experiment 1* to displace the cursor. There were two conditions for these trials, flexor mechanical disturbances with leftward visual disturbances and extensor mechanical disturbances with rightward mechanical disturbances, which were coupled with flexor and extensor background loads, respectively. This trial type consisted of 50 trials in total, 25 trials for each condition.

Importantly, in these tasks, the cursor and not direct hand position was provided to the participants to maximize the influence of cursor feedback on the motor system. This ensured participants could not simply ignore the visual feedback. This only impacted how participants corrected for the V [v-shift] trials in *experiment 1* and PV [v-conflict] trials in *experiment 2* due to the dissociation of the position of the cursor and hand.

### Muscle Recordings

Electromyography (EMG) were recorded using bipolar surface electrodes (DE-2.1 EMG Sensor, Delsys, Natick, MA) from upper-limb muscles involved with flexion or extension at the elbow or shoulder (6, 23): the posterior deltoid (DP), the lateral head of the triceps (TLAT), the brachioradialis (BR), and pectoralis major (PM). Before the electrode placement on the muscle belly, the skin was cleaned and lightly abraded with cotton gauze and rubbing alcohol. The electrode contacts were covered with conductive gel. A ground electrode was placed on the lateral side of the elbow. EMG signals were amplified (Bagnoli-8 EMG System, Delsys, Natick, MA) and digitized at 1 KHz.

### Data Analysis

#### Filtering.

All data were aligned on the disturbance onset (either mechanical or visual). Kinematic data (hand positions and velocities) were low-pass filtered (20 Hz, 0-lag 6th-order Butterworth). All EMG signals were band-pass filtered (25–250 Hz, 0-lag 6th-order Butterworth), full-wave rectified, and then low-pass filtered (50 Hz, 0-lag 6th-order Butterworth) to reduce noise (10, 24–26).

#### Correction of video display latency.

With any video display system, there are latencies between when a command is sent to display an image and the time at which the corresponding image is actually displayed to the participants. We estimated these latencies by logging the time from when a visual disturbance (e.g., cursor shift) was commanded by the software to when the video card began processing that command. This time difference includes latencies from video card asynchrony and wait time for Vsync pulses. In addition, another 17 ms was summed for every trial to account for the refresh cycle of the display. Latencies were estimated for each trial (43.39 ± 0.86 ms, mean ± SD). In the results, we report both when data are “aligned on the visualization onset,” as well as when data are “aligned on the mechanical disturbance onset.”

#### Muscle activity.

Data of BR muscle from two participants (1 subject each in *experiments 1* and *2*) were excluded from the offline analysis because of no detectable stretch responses. For individual trials, we excluded trials if *1*) there was abnormal preparatory activity, in which the amplitude of EMG signals in the preparation period (i.e., the period when participants held at the start position before the target appearance) exceeded >3 SD from the mean amplitude of all trials and *2*) there was apparent motion artifacts such as a large spike at the mechanical disturbance onset. In this screening process, 1.06%, 0.98%, 0.94%, and 1.10% of trials were excluded in *experiment 1* for DP, PM, TLAT, and BR, respectively. In *experiment 2*, 1.27%, 0.93%, 1.43%, and 2.04% of trials were excluded for DP, PM, TLAT, and BR, respectively. The EMG signal of each muscle was normalized to its mean response during the preparation period in the start position when the muscle actively countered a 1 N·m background load. In all cases, the normalization window was a length of 100 ms (27). The average EMG signal of baseline trials was subtracted from mechanical or visual disturbance trials for each subject (ΔEMG). For trials of mechanical disturbance with visual feedback and trials of visual disturbances, the average EMG signal of baseline trials with visual feedback was subtracted. Correspondingly, for trials of mechanical disturbance without visual feedback (*experiment 1*), the average EMG signal of baseline trials without visual feedback was subtracted. To compare the difference in muscle responses between trial types, EMG time series were binned into epochs reflecting two visual feedback responses (V1, 90–130 ms; V2, 140–180 ms; Ref. 10) in which the first phase of the motor response is independent for each sensory modality followed by a second phase in which the motor response reflects an integrated motor response (10, 19). We selected the 140–180 ms window instead of 120–180 ms in the original study (10) to match the duration of the two windows.

#### ROC analysis.

We used a receiver operating characteristic (ROC) technique (28) to determine when *1*) the muscle activity was reliably different between the two directions of mechanical or visual disturbances and *2*) EMG responses caused by mechanical disturbances were reliably different between experimental conditions. For each time step (1 ms), we generated an ROC curve and calculated the area under the curve (AUC) representing the probability of discrimination between the corresponding EMG data. First, we generated an ROC curve for the EMG time series averaged across trials for each participant. Normalized EMG traces were smoothed with a zero-lag 5-ms moving average filter before calculation of the ROC curve to reduce variability. AUC values of 0 and 1 indicate perfect discrimination, whereas a value of 0.5 indicates that the two distributions are fully overlapping and will not be discriminated with each other. Second, we determined that the muscle activity was reliably different when the AUC surpassed a threshold of 0.75 for 10 ms consecutively. Finally, the “knee” was found to determine onset by regressing back to the last timepoint where the AUC value crossed 0.50 (22). In the current study, to improve the quality of onset detection, we searched a local minimum nearest before the time the AUC crossed 0.50.

### Statistical Analysis

Statistical tests were performed for each muscle independently using MATLAB 2018a (MathWorks Inc., Natick, MA). In *experiment 1*, we performed a two-way repeated-measures ANOVA that included trial type (i.e., P [no-v] and V [v-shift] or PV [v-align]-P [no-v] and V [v-shift]) and time epoch (V1 and V2) as factors. In addition, to check the changes in muscle responses across trials, we also used a two-way repeated-measures ANOVA with trial bin (i.e., first 5 trials and last 5 trials) and time epoch (V1 and V2) as factors. If a significant interaction effect was found, we performed a post hoc Bonferroni test for each time epoch. In *experiment 2*, we performed a two-way repeated-measures ANOVAs that included trial type (i.e., PV [v-align] and PV [v-conflict]) and time epoch (V1 and V2) as factors, and trial bin (i.e., first 5 trials and last 5 trials) and time epoch (V1 and V2) as factors. In all comparisons, significance level was set at 0.05.

We also ensured our ANOVA results were not sensitive to any outliers in the data by rerunning the analysis using a robust ANOVA (29). With a robust ANOVA, a percentage (trim level) of data points are trimmed that are from both ends of the data distribution. For our data, we used a trim level of 10% and used a Winsorized approach where trimmed values were not discarded but set to the smallest or largest values where appropriate. Thus, the means used to compute the ANOVA statistics were the trimmed means.

### Model

We developed an optimal control model to quantify how muscle responses should be influenced by visual and proprioceptive feedback considering the influence of factors such as noise and sensory delays (18). The model describes the hand trajectory of reaching movements. The model includes a viscous torque opposing and proportional to the joint velocity with damping factor *G* = 0.15 N·s·m^−1^, corresponding to the linear approximation of viscosity at the elbow joint in humans (30). The equation of motion was coupled with a first order, low-pass filter linking motor commands captured in the control input *u*, with the controlled force as a linear model of muscle dynamics. The rise time of the muscle force in response to changes in control input was τ = 66 ms (31). Thus, the state variables include the cursor position (*p*), the hand position (*h*), the velocity (*v*), and the forces (*F_MC_*) generated from the control vector (*u*). These variables were augmented with the external force (*F_E_*, not controllable) used to simulate the external disturbance. Finally, the state vector was augmented with the target coordinate that will be necessary for the control problem. With these definitions, the continuous differential equation of the system is as follows:

(*3*) $$m\dot{v}=-Gv+F_{MC}+F_{E}$$

$$\text{τ}{\dot{F}}_{MC}=u-F_{MC}$$

$${\dot{F}}_{E}=0.$$

Defining the target coordinate by p*, the vector representing the state of the system is *x* = [*p*,*h*,*v*,*F_MC_*,*F_E_*,p*]*^T^*. The dynamics of the external torque from the point of view of the controller corresponds to the assumption that it follows a step function (${\dot{F}}_{E}$ = 0). The continuous differential equation above was transformed into a discrete time system by using Euler integration with a time step of δ*t* = 10 ms. The discrete time system allows additive and signal-dependent noise. The state-space representation of the discrete-time control system is as follows:

(*4*) $$x_{t+1}=Ax_{t}+Bu_{t}+{\text{ξ}}_{t}+\in_{t}Cu_{t}$$

$$A=\left[\begin{array}{cccccc} 1 & 0 & \Delta t & 0 & 0 & 0\\ 0 & 1 & \Delta t & 0 & 0 & 0\\ 0 & 0 & 1-\frac{G\Delta t}{m} & \frac{\Delta t}{m} & \frac{\Delta t}{m} & 0\\ 0 & 0 & 0 & 1-\frac{\Delta t}{\text{τ}} & 0 & 0\\ 0 & 0 & 0 & 0 & 1 & 0\\ 0 & 0 & 0 & 0 & 0 & 1 \end{array}\right],\;B=\left[\begin{array}{c} 0\\ 0\\ 0\\ \frac{\Delta t}{\text{τ}}\\ 0\\ 0 \end{array}\right],$$

Where *m* is the mass (1 kg), ξ*_t_* is an additive multivariate Gaussian noise with zero-mean and known covariance matrix, *C* is a scaling matrix, and ∈*_t_* is a zero mean and unit variance Gaussian noise. To include feedback delays into the model, we augmented the system with past states and made the most delayed state observable by the controller. Using δ*t_p_* and δ*t_v_* to designate the proprioceptive and visual delays in number of sample times, the augmented state vector is as follows (superscript *T* represents the transpose operator):

(*5*) $$x={\left[x^{T}{}_{t},x^{T}{}_{t-1},\ldots ,x^{T}{}_{t-\text{δ}t_{p}},\ldots ,x^{T}{}_{t-\text{δ}t_{v}}\right]}^{T}.$$

The delay was set to 50 ms for proprioceptive feedback (6) and 90 ms for visual feedback (18).

**Figure 2.**
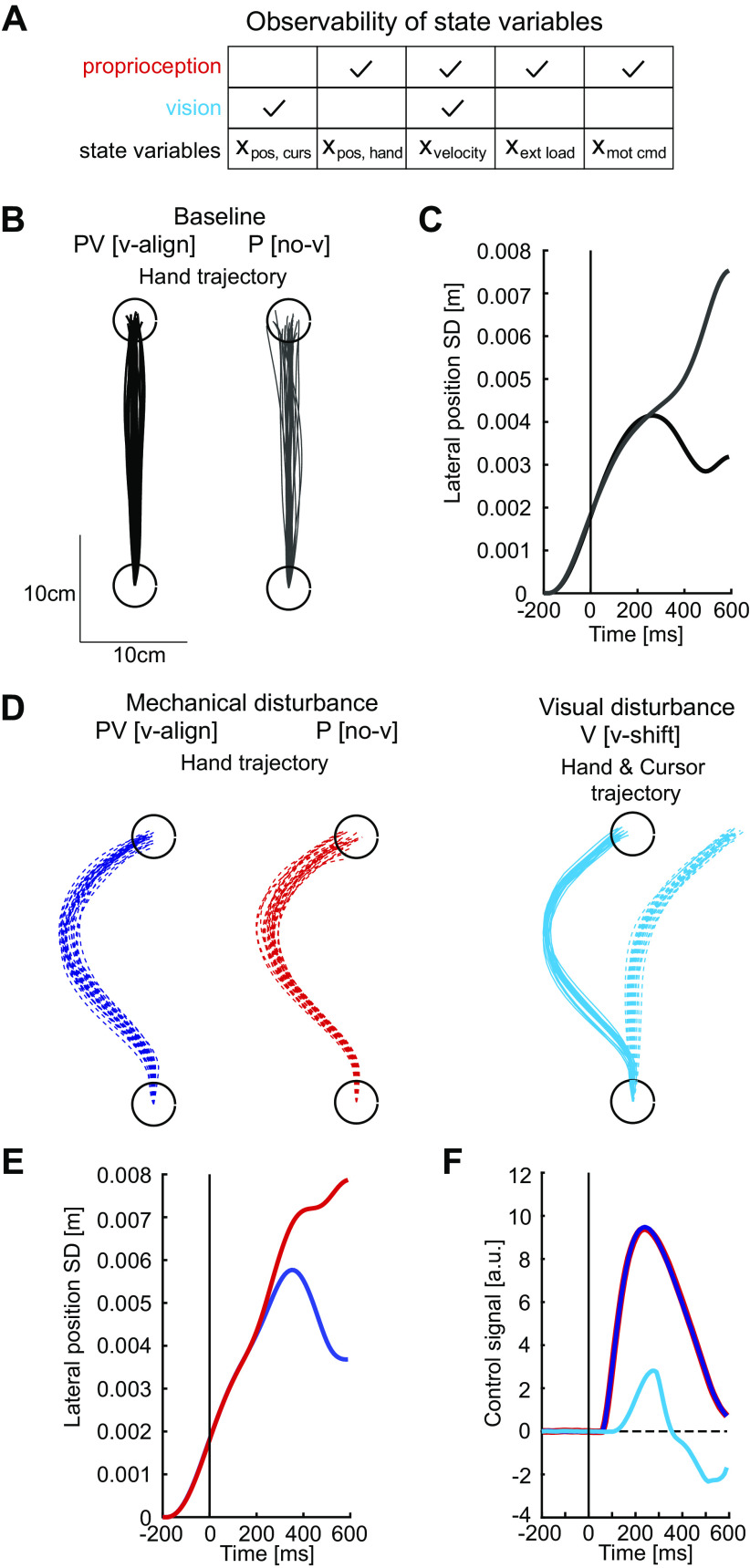
Simulation of the dynamic Bayesian model for *experiment 1*. *A*: table showing which state variables were directly observable by proprioception and vision. *B*: schematic description of predictions of hand trajectories in baseline trials. Black lines indicate the trials with visual feedback and gray lines indicates trials without visual feedback. Twenty-five simulated trajectories are shown. *C*: end point variability (standard deviation, SD) in lateral positions in baseline trials. The black line indicates trials with visual feedback, and the gray line indicates trials without visual feedback. Data were aligned on the timing when the hand had traveled approximately 3 cm from the start position. *D*: schematic description of predictions of hand trajectories in disturbance trials. Blue lines indicate the trial type of mechanical disturbance with aligned visual feedback, red lines indicate mechanical disturbance without visual feedback, and cyan lines indicate visual disturbance with cursor shift. The thick colored line in the *right* panel indicates average of cursor trajectories. *E*: end point variability (SD) in lateral positions in mechanical disturbance trials. Color coding was same as in *D*. Data were aligned on the mechanical disturbance onset (mechanical disturbance trials) and the visualization onset (visual disturbance trials). *F*: prediction of time traces of control signals in the lateral direction. A control signal in the lateral direction approximates muscle activities to move the arm in that direction. Average of baseline control signals was subtracted. Data were aligned on the mechanical disturbance onset (mechanical disturbance trials) and the visualization onset (visual disturbance trials). Color coding was same as in *D*. ext load, Extensor load; mot com, motor command; [no-v], no visual feedback provided; P, mechanical disturbance only; pos, curs, cursor position; pos, hand, hand position; PV, mechanical and visual disturbance; V, visual disturbance only; [v-align], visual feedback present and aligned with hand; [v-shift], visual shift of cursor.

In our previous study, we assumed that proprioception and vision updated the same state variables (18). In contrast, for the current model to resemble human performance, we assumed proprioception and vision only partially overlapped in the state variables that they observed (Fig. 2*A*). Specifically, we assumed that the cursor position could only be directly observed by visual feedback. This was necessary as the behavioral goal of the controller and human participants was to drive the cursor to the goal and not a weighted average of the position signaled by visual and proprioceptive feedback. This distinction was clearly apparent on trials where the cursor position was not aligned with the felt hand position (V[v-shift], PV[v-conflict]), as participants drove the cursor to the goal. We also assumed the velocity was directly observable by vision.

From proprioception, we assumed that it could observe the hand velocity, external forces, and applied motor commands, and thus did not directly observe the cursor position. However, despite not directly observing the cursor’s position, proprioceptive feedback could still be used to update the estimate of the cursor’s position through the use of an internal model. For example, changes in velocity detected by proprioceptive feedback updates the estimate of the limb’s velocity which in turn can be used to update the cursor’s position on the next time step using a forward model. Thus, proprioception can still update state variables that are only directly observable by vision.

To accommodate this, we defined the observation matrix *H* as follows:

(*6*) $$H=\left[\begin{array}{ccc} O_{n},\ldots , & H_{p},\ldots , & O_{n}\\ O_{n},\ldots , & O_{n},\ldots , & H_{v} \end{array}\right].$$

With *O_n_* representing the zero square matrices of size *n*, respectively, and where *H_p_* and *H_v_* are the submatrices defining the state variables that proprioceptive and visual feedback could directly observe and were defined as

$$H_{p}=\left[\begin{array}{cccccc} 0 & 0 & 0 & 0 & 0 & 0\\ 0 & 1 & 0 & 0 & 0 & 0\\ 0 & 0 & 1 & 0 & 0 & 0\\ 0 & 0 & 0 & 1 & 0 & 0\\ 0 & 0 & 0 & 0 & 1 & 0\\ 0 & 0 & 0 & 0 & 0 & 1 \end{array}\right]$$

$$H_{v}=\left[\begin{array}{cccccc} 1 & 0 & 0 & 0 & 0 & 0\\ 0 & 0 & 0 & 0 & 0 & 0\\ 0 & 0 & 1 & 0 & 0 & 0\\ 0 & 0 & 0 & 0 & 0 & 0\\ 0 & 0 & 0 & 0 & 0 & 0\\ 0 & 0 & 0 & 0 & 0 & 1 \end{array}\right]$$

The *H_p_* matrix in the first block-row of *Eq. 6* corresponds to the time step *t* − δ*t_p_*, and the *H_v_* matrix of the second block-row corresponds to the time step *t* − δ*t_v_*. A simulation of mechanical disturbance without visual feedback (i.e., proprioceptive feedback only) was performed by using only the first block-row of the Matrix *H*. By defining ω*_t_* as the additive noise composed of the proprioceptive and visual noise as follows:

(*7*) $${\text{ω}}^{T}{}_{t}=\left[{\text{ω}}^{T}{}_{p,t},{\text{ω}}^{T}{}_{v,t}\right],$$

the feedback equation can be written in the following way:

(*8*) $$y_{t}=Hx_{t}+{\text{ω}}_{t}.$$

The covariance matrix of ω*_t_* is defined as Σ_ω_, which is a block-diagonal matrix composed by covariance matrices of proprioceptive (Σ*_p_*) and visual (Σ*_v_*) feedbacks:

(*9*) $$\Sigma_{\text{ω}}=\text{diag}\left[\Sigma_{p},\Sigma_{v}\right].$$

Then, *Eq. 8* can be rewritten as the following form.

(*10*) $$y_{t}=\left[\begin{array}{c} y_{t,\text{prop}}\\ y_{t,\text{vis}} \end{array}\right]=\left[\begin{array}{c} x_{t-\text{δ}t_{p}}\\ x_{t-\text{δ}t_{v}} \end{array}\right]+\left[\begin{array}{c} {\text{ω}}^{T}{}_{p,t}\\ {\text{ω}}^{T}{}_{v,t} \end{array}\right].$$

Background assumptions of this feedback equation are discussed in detail in Crevecoeur et al. (18). As with conventional optimal feedback control, dynamic Bayesian estimation uses Kalman filtering, which weighs sensory feedback and priors to deduce a maximum-likelihood estimate of the system state. Let ${\hat{x}}_{t}$ represent the expected value of the present state given the past estimate and *u_t_* represent control input, the computation of the prior is obtained by simulating the system dynamics as follows:

(*11*) $${\hat{x}}^{\text{prior}}{}_{t+1}=A{\hat{x}}_{t}+Bu_{t}+{\text{ξ}}_{t},$$

where ξ*_t_* denotes variability in the prediction with a zero-mean Gaussian noise with covariance Σ_INTERNAL_. Now the estimated state can be expanded as follows:

(*12*) $${\hat{x}}_{t+1}={\hat{x}}^{\text{prior}}{}_{t+1}+K_{p}\left(y_{t,\text{prop}}-H_{v}\;{\hat{x}}^{\text{prior}}{}_{t}\right)+K_{v}\left(y_{t,\text{vis}}-H_{p}\;{\hat{x}}^{\text{prior}}{}_{t}\right)$$

(*13*) $${\hat{x}}_{t+1}={\hat{x}}^{\text{prior}}{}_{t+1}+K_{p}\left(y_{t,\text{prop}}-{\hat{x}}^{\text{prior}}{}_{t-\text{δ}t_{p}}\right)+K_{v}\left(y_{t,\text{vis}}-{\hat{x}}^{\text{prior}}{}_{t-\text{δ}t_{v}}\right).$$

*y_t_*_,prop_ and *y_t_*_,vis_ represent the block components of *y_t_* corresponding to proprioceptive and visual signals. *K* corresponds to the Kalman gains that are computed offline. Note, this equation highlights how the Kalman filter accounts for delays when updating the current estimate of the limb. Rather than estimating the current state from sensory feedback by simply weighting proprioceptive and visual feedback that are at different latencies (i.e., ${\hat{x}}_{\text{est}}=\;w_{\text{prop}}y_{t,\text{prop}}+w_{\text{vis}}y_{t,\text{vis}}$) as typical of classic multisensory integration, the Kalman filter instead compares feedback for each sensory modality with the prior estimate of the limb state at the appropriate time delay for the sensory modality (e.g., $y_{\text{prop}}-{\hat{x}}^{\text{prior}}{}_{t-\text{δ}t_{p}}$).

Noise parameters were defined following standard approaches: the covariance matrix of the additive noise was defined as Σ_ξ_ = σ^2^BB*^T^* (σ = 0.066). The motor noise was composed of two terms: an additive term plus a control-dependent term with principal axis aligned with the direction of the control vector with variance (0.015)^2^ × *u* and a secondary axis orthogonal to the control vector with variance (0.005)^2^ × *u* (6, 32). For the sensory noise, we fixed the covariance matrix of the proprioceptive noise to Σ*_p_* = 10^−5^ × *I_n_*, where *n* is the dimension of the state vector and *I_n_* is the identity matrix of size *n*. The variance of the proprioceptive noise was defined to satisfy Σ*_p_* = 2.3 × Σ*_v_* (33). The value of 2.3 corresponds to weighting vision by 70% and proprioception by 30%. The remaining noise to be defined is the internal noise affecting the computation of the prior estimate. We chose Σ_INTERNAL_ = 0.8 × 10^−6^ at each time step (18).

The cost-function to minimize is a quadratic penalty on position error and control, which is written as follows:

(*14*) $$J\left(p,u\right)=\overset{N}{\underset{t=1}{\sum }}{\left(p_{t}-p^{*}\right)}^{2}+Ru_{t}{}^{2},$$

with *R* = 10^−6^. The optimal control policy is a linear function of the following estimated state:

(*15*) $$u_{t}=L{\hat{x}}_{t},$$

where *L* denotes the optimal feedback gains. The computation of the optimal feedback gains and Kalman gains are described thoroughly in the study by Todorov (34), which can handle systems that are partially observable.

For modeling of trials with only a visual disturbance (V [v-shift]), we altered position and velocity components in the visual feedback of the observation matrix (*y_t_*) with the average of position components and the average of velocity components of state vectors simulated for trials with mechanical disturbance with visual feedback (PV [v-align]).

To predict variability in hand position during movements, we generated 25 simulated trajectories per cycle to calculate a standard deviation of lateral positions at each timepoint and repeated it for 1,000 cycles. Data and model code for the simulation are available at https://www.github.com/KevinCross/DynamicBayesianModel.

## RESULTS

### Experiment 1

#### Dynamic Bayesian model predictions.

We developed an optimal control model to quantify how muscle responses should be influenced by visual and proprioceptive feedback while considering the influence of factors such as noise and sensory delays similar to our previous model (18). However, one aspect by which this model differs from our previous implementation is that proprioception and vision could only directly observe a subset of state variables (Fig. 2*A*). Specifically, we assumed proprioception could observe the hand position, velocity, external forces, and the motor commands, whereas vision could observe the cursor position and the velocity. We found this resulted in a better match to human performance in the task (See *Human Performance*); however, our main conclusions were not affected had proprioception and vison observed all state variables (data not shown).

On average, simulated hand trajectories on baseline reaches were qualitatively similar whether or not we provided the model with visual feedback (Fig. 2*B*). Note, we did not reoptimize the feedback or Kalman gains when we removed visual feedback and simply zeroed the appropriate entries in the observation matrix (*H*) on those trials. When visual feedback was present (PV[v-align]), the variability in the simulated lateral hand position increased to the midway point of movement and then decreased to the end of movement (Fig. 2*C*). When visual feedback was not present (P[no-v]), variability was similar up to the midway point of movement, but then continued to increase toward the end of movement. Thus, visual feedback was not simply ignored by the model consistent with our previous findings (18).

When mechanical disturbances were applied to the limb, the model generated a rapid motor correction to oppose the applied load and attain the spatial goal (Fig. 2*D*). In this situation, hand trajectories were qualitatively similar whether visual feedback was available or not during reaching (blue vs. red traces). With both sensory modalities, trial-to-trial variability in lateral hand position increased past the midway point of the movement and then decreased to the end of movement (Fig. 2*E*). A similar pattern was observed when there was no visual feedback, although the variability in lateral hand position continued to increase toward the end of the movement.

For visual disturbances, we used a gradual shift of the cursor position so that we could compare motor corrections for mechanical versus visual disturbances (Fig. 2*D*, cyan traces). Here, the cursor followed a trajectory similar to the trajectory the cursor followed on the mechanical load trials (V [v-shift] trials). Note, the visual disturbance was signaled to the model by altering the visual feedback of the cursor position. Visual feedback of the velocity was also altered, and thus the controller had to estimate the velocity from conflicting sensory feedback. Importantly, these trials generated the same kinematic visual error that the controller observed on PV [v-align] trials without the accompanying proprioceptive error. For these trials, the model predicted corrections that began at approximately the midpoint of the movement.

Next, we examined how the control signal differed for corrective responses when proprioceptive and visual feedback about the error were provided versus when only one modality signaled the error (Fig. 2*F*). When only proprioceptive feedback was disturbed by the mechanical load (P [no-v]) the control signal increased from baseline starting at 50 ms postdisturbance, reflecting the proprioceptive delay in the model. Similarly, when only visual feedback was disturbed using a kinematic error, the control signal increased from baseline starting at ∼90 ms and was relatively small reflecting in part that there was no applied load that had to be counteracted (Fig. 2*F*). In the 90–130 ms epoch, the PV [v-align] was 3.54 N which was less than the simple addition of the P [no-v] (3.58 N) and V [v-shift] (0.023 N) trials, although this difference was small (additive model: 3.61 N). Differences were more noticeable in the 140–180 ms epoch with output during PV [v-align] trials at 7.04 and the additive model at 7.62 N (P [no-v] = 7.09 N, V [v-align] = 0.52 N). It is important to stress that the negligible change in motor response observed when vision is available is the signature of a multisensory integration mechanism that accounts for sensory delays and incorporates a dynamics model of limb. Within the model, proprioceptive feedback updates several state variables related to the forces acting on the limb and the velocity of the limb. Through the use of a forward model, these state variables can then be used to infer the updated internal estimate of the cursor position. As a result, by the time the visual feedback reaches the controller, the state estimate has already been largely updated by proprioception. Thus, the subadditive response to proprioceptive and visual feedback is an emergent and specific property of the dynamic Bayesian integration model.

We also performed an additional sensitivity analysis for the model by varying the noise and delay of visual feedback (Supplemental Fig. S1; see https://doi.org/10.5281/zenodo.5725741). We tested Σ*_p_* = 4.6 × Σ*_v_* and Σ*_p_* = 1.15 × Σ*_v_* (i.e., double and half of the original value of 2.3) and the delay of 80 ms and 100 ms (i.e., approximately ±10% of the original value of 90 ms). The results showed qualitatively similar responses in all types of disturbance and the range of increase in the motor response when vision is available was <0.1% to 0.48%. Critically, the additive model was always larger with a range of 5.2% to 11.8%.

#### Human performance.

The results from the dynamic Bayesian model make predictions that are distinct from the two-stage model about how early corrective responses should combine proprioceptive and visual feedback. The dynamic Bayesian model predicts that proprioceptive and visual feedback should combine in a subadditive manner. In contrast, the two-stage model predicts that proprioceptive and visual feedback should combine in an additive manner for the earliest correction. Next, we explored these two hypotheses in humans performing goal-directed reaches.

**Figure 3.**
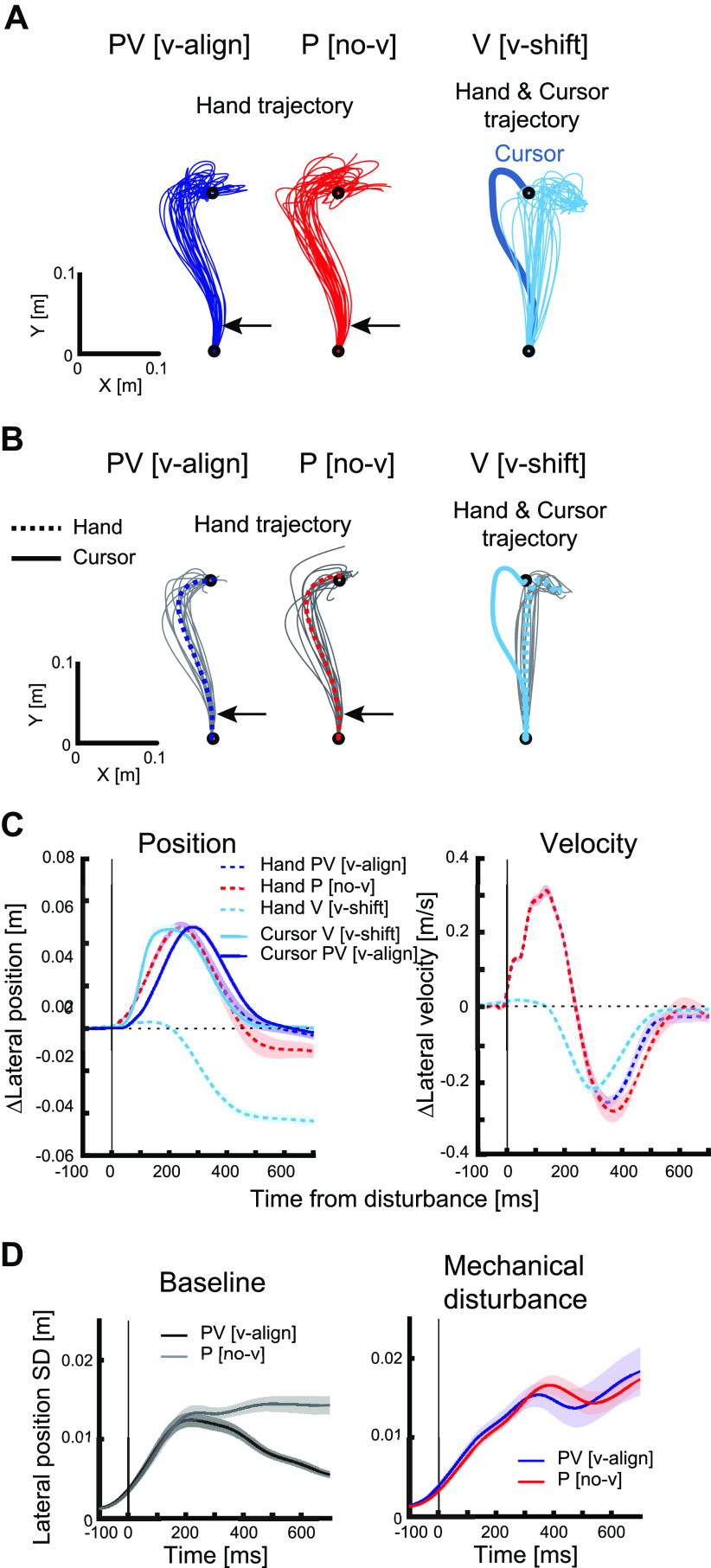
Kinematics during *experiment 1*. *A*: hand and cursor trajectories in an exemplar participant. Blue lines indicate hand trajectories across trials for mechanical disturbances with aligned visual feedback, red lines indicate trials for mechanical disturbances without visual feedback, and cyan thin lines indicate trials for visual disturbances with cursor shift. The thick line in the *right* panel shows the trial-averaged cursor trajectory. Black arrows indicate the timing of mechanical loads. *B*: hand and cursor trajectories in all participants. Gray lines indicate trial-averaged trajectories for each participant, dotted colored lines indicate across-participants average of hand trajectories, and the solid colored line in the *right* panel indicates across-participants average of cursor trajectories. *C*: lateral position and velocity changes for extensor disturbances. Each line indicates across-participants average in each trial type and shaded areas indicate mean ±1 SE. Average of baseline trials was subtracted. Dashed lines indicate hand position/velocity and solid lines indicate cursor position. Blue lines indicate the trial type of mechanical disturbance with aligned visual feedback (PV [v-align]), red lines indicate mechanical disturbance without visual feedback (P [no-v]), and cyan lines indicate visual disturbance with cursor shift (V [v-shift]). Data of hand traces are aligned on the mechanical disturbance onset (PV [v-align] and P [no-v]) or the visualization onset (V [v-shift]; i.e., the time when the visual disturbance started on the monitor after correcting the visualization delay). Data of cursor traces are aligned on the time when the disturbance signal is commanded (the visualization delay was not corrected). *D*: across-participants average of end point variability (SD) in lateral positions. *Left*: the black line indicates baseline with visual feedback, and the gray line indicates baseline without visual feedback. Data were aligned on the timing when the hand had traveled 3 cm from the start position. *Right*: the blue line indicates the trial type of mechanical disturbance with aligned visual feedback, and the red line indicate mechanical disturbance without visual feedback. Data were aligned on the mechanical disturbance onset. Shaded areas indicate mean ±1 SE.

In general, human motor corrections were qualitatively similar to the dynamic Bayesian model with regards to the influence of visual and/or proprioceptive feedback on motor corrections. We performed an experiment in which participants reached to a target including trials with and without visual feedback, as well as with either mechanical or visual disturbances (Fig. 1*B*). Average hand trajectories in mechanical disturbance trials were similar with (PV [v-align]) and without (P [no-v]) aligned visual feedback (Fig. 3, *A* and *B*). Hand position and velocity were also similar between these two trial types until ∼400 ms postdisturbance (Fig. 3*C*).

The magnitude of the lateral deviation for the visual shifts of the cursor (V [v-shift], Fig. 3*C*, *left*) was similar to the hand lateral deviations for mechanical disturbances (PV [v-align] and P [no-v]). In Fig. 3*C*, the peak lateral shift for PV [v-align] was 4.85 ± 0.25 cm (mean ± SE), P [no-v] was 4.86 ± 0.24 cm, and V [v-shift] was 4.69 ± 0.23 cm. However, the cursor shift in V [v-shift] is clearly faster than the cursor motion induced by mechanical loads applied to the arm (PV [v-align]).

In baseline trials, movement variability initially increased after movement onset, but decreased toward the end of movement when visual feedback was available (Fig. 3*D*). Without visual feedback, movement variability increased to peak movement speed and then remained higher to the end of movement, as qualitatively observed for the dynamic Bayesian model. When mechanical disturbances were applied during reaching, movement variability grew continuously throughout movement, and remained high to the end of movement. Unlike the simulations, whether vision was present or absent did not lead to a reduction in the variability near the end of movement.

**Figure 4.**
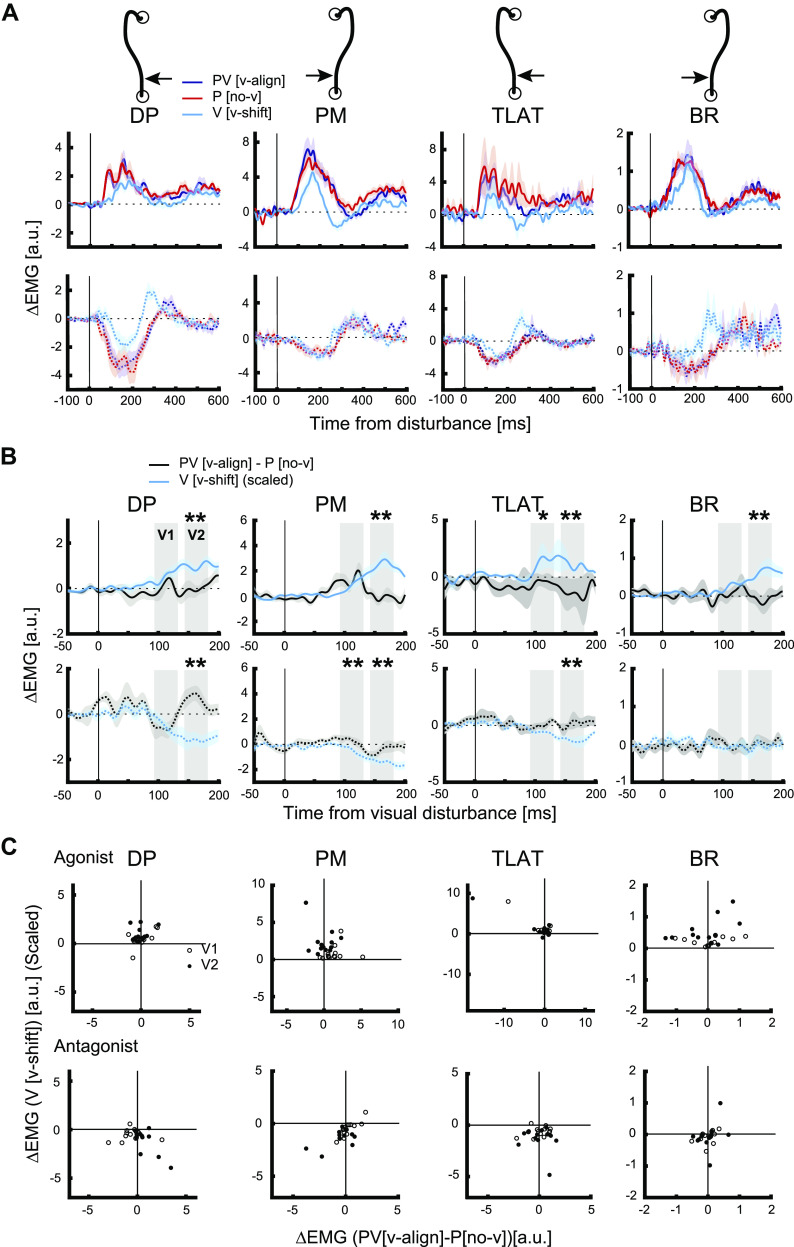
Muscle responses during *experiment 1*. *A*: across-participants average of muscle activities. Average of baseline trials was subtracted. Electromyography (EMG) signals were normalized to its mean response during the preparation period in the start position when the muscle actively countered a 1 N·m background load. Blue lines indicate the trial type of mechanical disturbance with aligned visual feedback, red lines indicate mechanical disturbance without visual feedback, and cyan lines indicate visual disturbance with cursor shift. Solid lines indicate agonist disturbance (extensor load/leftward cursor shift for DP and TLAT and flexor load/rightward cursor shift for PM and BR) and dotted lines indicate disturbances in the opposite direction. Data were aligned on the mechanical disturbance onset (mechanical disturbance) and the visualization onset (visual disturbance with cursor shift). *B*: across-participants average muscle response of the trials with mechanical disturbance with visual feedback in which the mean muscle response for the mechanical disturbance with no visual feedback was subtracted (black lines, “PV [v-align]–P [no-v]”), and the across-participants average muscle response of the trials with visual disturbance with cursor shift that was scaled by the ratio between the cursor shift amplitude of PV [v-align] and V [v-shift] during 0–90 ms after the disturbance onset (cyan lines, “V [v-shift]”). The gray boxes in each panel indicate V1 (90–130 ms) and V2 (140–180 ms) epochs. Data were aligned on the visualization onset. Asterisks indicates significant difference in mean muscle activities between “PV [v-align]–P [no-v]” and “V [v-shift]” during V1 or V2 epochs (*P < 0.05 and **P < 0.01). *C*: scatter plots of muscle responses of the trials with mechanical disturbance with visual feedback in which the mean muscle response for the mechanical disturbance with no visual feedback was subtracted (“PV [v-align]–P [no-v]”) and visual disturbance with cursor shift (“V [v-shift]”) which was scaled in the same manner as in *B*. Each dot indicates each participant, and open circles indicate V1 and filled circles indicate V2 epochs. The *top* row shows responses for agonist disturbance and the *bottom* row shows responses for antagonist disturbance. Note, data points that resided outside the bounds of the axes had their values denoted in text and placed on the axes boundary. a.u., Arbitrary units; BR, brachioradialis; DP, posterior deltoid; PM, pectoralis major; TLAT, lateral head of the triceps.

Figure 4*A* displays the activity of arm muscles for mechanical disturbances with and without visual feedback. The first row indicates muscle activities during disturbances in which each muscle acted as an agonist (i.e., stretched), whereas the second row indicates disturbances in which each muscle acted as an antagonist. The amplitude indicates the increase/decrease in muscle activities compared with baseline trials. When the muscle is an agonist, the motor responses for a mechanical disturbance without vision (P [no-v]) is consistently larger than that observed for a similar-sized visual disturbance (V [v-shift]). We quantified the motor responses to those two types of disturbances in two time windows (V1, 90–130 ms; V2, 140–180 ms). For agonist disturbances (i.e., extensor load or leftward cursor shift for DP and TLAT, flexor load or rightward cursor shift for BR and PM), a two-way repeated-measures ANOVA (2 trial types × 2 epochs) found a significant main effect of trial type in all muscles (DP, *F*_1,12 _= 97.50, *P* < 0.001; PM, *F*_1,12 _= 81.39, *P* < 0.001; TLAT, *F*_1,12_ = 30.58, *P* < 0.001; BR, *F*_1,11_ = 69.98, *P* < 0.001). The main effect of epoch was significant in all except for TLAT (DP, *F*_1,12_ = 31.42, *P* < 0.001; PM, *F*_1,12 _= 64.53, *P* < 0.001; TLAT, *F*_1,12 _= 0.46, *P* = 0.513; BR, *F*_1,11 _= 50.11, *P* < 0.001). The interaction effect (trial type × epoch) was not significant in all muscles (DP, *P* = 0.667; PM, *P* = 0.186; TLAT, *P* = 0.228; BR, *P* = 0.115). A post hoc test indicated a significant difference between P [no-v] and V [v-shift] in V1 (90–130 ms) for all muscles and in V2 (140–180 ms) for all except for TLAT (DP, V1 [*P* < 0.001], V2 [*P* < 0.001]; PM, V1 [*P* < 0.001], V2 [*P* = 0.001]; TLAT, V1 [*P* = 0.003], V2 [*P* = 0.053]; BR, V1 [*P* < 0.001], V2 [*P* = 0.004], Bonferroni corrected). For antagonist disturbances (i.e., flexor load or rightward cursor shift for DP and TLAT, extensor load or leftward cursor shift for BR and PM), a significant main effect of trial type was found in all muscles (DP, *F*_1,12_ = 37.19, *P* < 0.001; PM, *F*_1,12 _= 5.62, *P* = 0.035; TLAT, *F*_1,12_ = 7.54, *P* = 0.018; BR, *F*_1,11 _= 18.84, *P* < 0.001), and the main effect of epoch was not significant in all except for PM (DP, *F*_1,12_ = 2.11, *P* = 0.172; PM, *F*_1,12 _= 37.35, *P* < 0.001; TLAT, *F*_1,12 _= 2.04, *P* = 0.178; BR, *F*_1,11_ = 0.56, *P* = 0.471). The interaction effect was not significant in all except for PM (DP, *P* = 0.066; PM, *P* = 0.020; TLAT, *P* = 0.089; BR, *P* = 0.547). A post hoc test indicated a significant difference between P [no-v] and V [v-shift] in V1 in all except for BR, and in V2 only for BR (DP, V1 [*P* = 0.001], V2 [*P* = 0.083]; PM, V1 [*P* = 0.023], V2 [*P* = 1.000]; TLAT, V1 [*P* = 0.042], V2 [*P* = 1.000]; BR, V1 [*P* = 0.140], V2 [*P* = 0.029]).

As noted earlier and displayed in Fig. 3*C*, the lateral motion of the cursor (V [v-shift] trials) is clearly faster than the deviation of the hand for the mechanical disturbances (PV [v-align] trials). Thus, it would not be surprising that the motor response in the visual-only disturbance would be larger than the potential contribution of vision during the mechanical disturbances. To account for this effect, we scaled down the muscle response of V [v-shift] by quantifying the ratio of the average cursor lateral shift of PV [v-align] to that of V [v-shift] from 0 to 90 ms after the visual disturbance onset. We found cursor motion was 1.36/1.56 (leftward/rightward disturbances) times greater for the V [v-shift] trials. Thus, we correspondingly reduced the size of the muscle response by 73.4/64.9% for V [v-shift] trials in all further diagrams and analyses. Note, our results were not affected if we calculated the scaling factor for each individual separately or at the group level, nor if we varied the scaling factor by ±10%.

Our key test is whether the contribution of vision to the motor response during a mechanical disturbance is less than that observed for a visual disturbance on its own. Thus, the mean muscle response for the mechanical disturbance with no visual feedback was subtracted from each trial in which the mechanical disturbance included visual feedback (PV [v-align]-P [no-v]). These trials were then realigned to account for the delays in the visual display and compared with the muscle response for the visual-only disturbances (Fig. 4*B*). The prediction by the dynamic Bayesian model is that the change in muscle response to V [v-align] will be greater than the muscle response for PV [v-align]-P [no-v].

Indeed, changes in muscle responses for V [v-align] trials were generally larger than for PV [v-align]-P [no-v] (Fig. 4*B*). This was particularly prevalent in the V2 epoch for all muscle samples. Similar trends were observed when looking at individual participants as shown in the scatter plots comparing V [v-align] and PV [v-align]-P [no-v] in Fig. 4*C*. For V [v-align] in the V2 epoch, there is a noticeable bias toward values above zero for the agonist direction (*top* row) and a bias toward values below zero for the antagonist direction (*bottom* row). In contrast, there is less of a bias for PV [v-align]-P [no-v] trials.

We quantified these observations for agonist disturbances using a two-way repeated-measures ANOVA (2 trial types × 2 epochs) and found a significant main effect of trial type in all muscles (DP, *F*_1,12_ = 34.44, *P* < 0.001; PM, *F*_1,12_ = 9.868, *P* = 0.009; TLAT, *F*_1,12_ = 36.56, *P* < 0.001; BR, *F*_1,11_ = 24.67, *P* < 0.001). The main effect of epoch was not significant in all muscles. The interaction effect (trial type × epoch) was significant in DP and PM (DP, *P* = 0.023; PM, *P* < 0.001). A post hoc test indicated a significantly smaller excitation for PV [v-align]-P [no-v] as compared with V [v-shift] in V1 for TLAT (*P* = 0.047, Bonferroni corrected) and V2 for all muscles (DP, *P* < 0.001; PM, *P* = 0.001; TLAT, *P* = 0.001; BR, *P* = 0.001). For antagonist disturbances, significant main effect of trial type was found in all except for BR (DP, *F*_1,12_ = 11.16, *P* = 0.006; PM, *F*_1,12 _= 40.60, *P* < 0.001; TLAT, *F*_1,12 _= 14.05, *P* = 0.003; BR, *F*_1,11_ = 0.99, *P* = 0.342). The main effect of epoch was significant in PM (*F*_1,12_ = 50.08, *P* < 0.001). The interaction effect (trial type × epoch) was significant in DP (*P* = 0.003). A post hoc test indicated a significantly smaller inhibition of PV [v-align]-P [no-v] as compared with V [v-shift] in V1 for PM (*P* = 0.006) and V2 for all except for BR (DP, *P* = 0.002; PM, *P* = 0.003; TLAT, *P* = 0.018; BR, *P* = 1.000). Collectively, these results indicate that V [v-shift] was greater than PV [v-align]-P [no-v] in the 140–180 ms epochs for all muscles but was only significantly greater in the 90–130 ms for two out of the eight muscle samples.

One concern is that several muscle groups had clear outliers (see Fig. 4*C* *top* row TLAT, values that are thresholded) that may have impacted our ANOVA. To alleviate this concern, we applied a robust ANOVA (see materials and methods) and found the exact same results. For agonist disturbances, the main effect of trial type was significant for each muscle (DP, *F*_1,12_ = 13.79, *P* = 0.002; PM, *F*_1,12 _= 7.0, *P* = 0.02; TLAT, *F*_1,12_ = 6.5, *P* = 0.02; BR, *F*_1,11_ = 7.15, *P* = 0.014). The interaction effect was significant in PM (PM, *P* = 0.001). A post hoc test indicated a significantly smaller excitation for PV [v-align]-P [no-v] as compared with V [v-shift] in V2 for all muscles (DP, *P* = 0.001; PM, *P* < 0.001; TLAT, *P* = 0.02; BR, *P* = 0.02).

For antagonist disturbances, significant main effect of trial type was found in all except for BR (DP, *F*_1,12_ = 5.45, *P* = 0.03; PM, *F*_1,12_ = 19.0, *P* = 0.001; TLAT, *F*_1,12_ = 16.6, *P* = 0.001; BR, *F*_1,11 _= 1.1, *P* = 0.31). The interaction effect was significant in DP (*P* = 0.006). A post hoc test indicated a significantly smaller inhibition of PV [v-align]-P [no-v] as compared with V [v-shift] in V2 for DP, PM, and TLAT (DP, *P* = 0.03; PM, *P* = 0.001; TLAT, *P* = 0.04). Finally, we investigated if the muscle response changed across trials by comparing the first and last five trials of each type of disturbance. We show the results for agonist disturbances here only. For the visual-only disturbance (V [v-shift]), a significant main effect of the trial bin (i.e., first/last 5 trials) was detected in a two-way repeated-measures ANOVA (2 trial bins × 2 epochs) in all muscles (DP, *F*_1,12 _= 8.43, *P* = 0.013; PM, *F*_1,12_ = 15.10, *P* = 0.002; TLAT, *F*_1,12 _= 11.04, *P* = 0.006; BR, *F*_1,11 _= 47.28, *P* < 0.001). A post hoc Bonferroni test indicated a significant decrease of muscle responses in V1 for DP ([*P* = 0.020]) and TLAT ([*P* = 0.043]), and V2 for PM ([*P* = 0.019]) and BR ([*P* < 0.001]). For mechanical disturbance with vision (PV [v-align]), significant main effect of the trial bin was found in TLAT (*F*_1,12 _= 11.27, *P* = 0.006) and BR (*F*_1,11_ = 40.17, *P* < 0.001). A post hoc Bonferroni test indicated a significant decrease of muscle responses in V1 for TLAT (*P* = 0.025) and BR (*P* = 0.045) and V2 for BR (*P* = 0.001). For mechanical disturbance without vision (P[no-v]), significant main effect of the trial bin was found for TLAT (*F*_1,12 _= 40.35, *P* < 0.001) and BR (*F*_1,11 _= 19.10, *P* = 0.001). A post hoc Bonferroni test indicated a significant decrease of muscle responses in V1 for TLAT (*P* = 0.001) and V2 for TLAT (*P* = 0.035) and BR (*P* = 0.004). Thus, some changes in the motor responses were observed from the beginning to the end of the experimental sessions that commonly reflected a decrease in the size of the motor response.

### Experiment 2

#### Dynamic Bayesian model predictions.

At this stage, we have demonstrated that proprioceptive and visual feedback are subadditively combined when responding to limb disturbances, consistent with the dynamic Bayesian model. However, an alternative possibility is that visual feedback is generally suppressed during limb disturbances. Such a mechanism would produce clear responses when each sensory modality is perturbed independently and a response similar to the one obtained after mechanical loads in the combined case. *Experiment 2* was designed as a control to assess this possibility by instructing participants to reach to a spatial goal when there were conflicting visual and mechanical disturbances (Fig. 1*D*). If visual feedback is suppressed for a mechanical disturbance, then we would observe similar responses for trials with conflicting sensory inputs as for trials with congruent sensory inputs.

**Figure 5.**
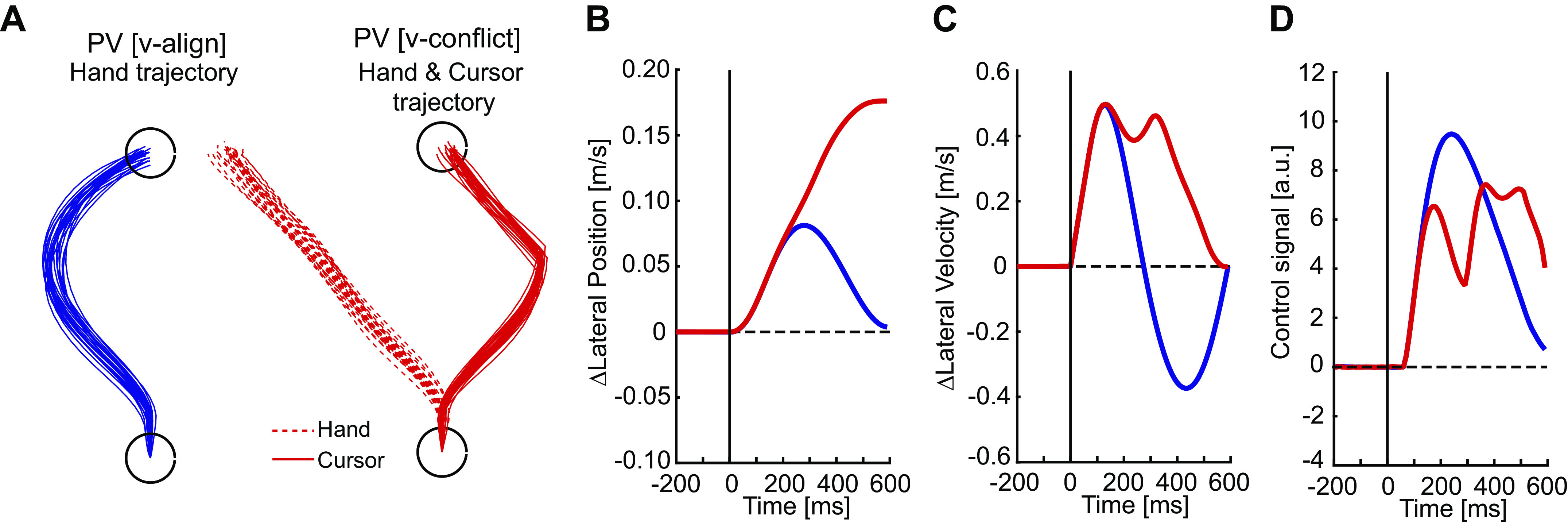
Simulation of the dynamic Bayesian model for *experiment 2*. *A*: schematic description of predictions of hand trajectories for the trial type of mechanical disturbance with aligned visual feedback (PV [v-align]; *left*), and the hand (dashed) and cursor (solid) trajectories in the trial type of mechanical and visual disturbance with conflicting visual feedback (PV [v-conflict]; *right*). *B*–*D* are the change in position, change in velocity, and the control signal averaged across trials. a.u., Arbitrary units.

First, we examined how the same dynamic Bayesian model used in *experiment 1* performed when vision and proprioception were in conflict. Note, we did not reoptimize the feedback and Kalman gains and instead used the same gains from *experiment 1*. Figure 5*A* shows the performance of the model after a load was applied when visual and proprioceptive feedback were aligned (blue; PV [v-align]) and when the cursor moved in the direction opposite of the hand (red; PV [v-conflict]). Trials in which feedback was aligned with the mechanical disturbance led to a corrective response to return to the spatial goal with peak lateral deviation occurring at ∼300 ms postdisturbance (Fig. 5, *B* and *C*). Lateral hand motion for trials in which visual feedback was in the opposite direction were initially similar up to peak lateral deviation, but then the hand moved more laterally to return the cursor toward the spatial goal. Correspondingly, there was a reduction in the control output for PV [v-conflict] that started at ∼90 ms (Fig. 5*D*).

#### Human performance.

**Figure 6.**
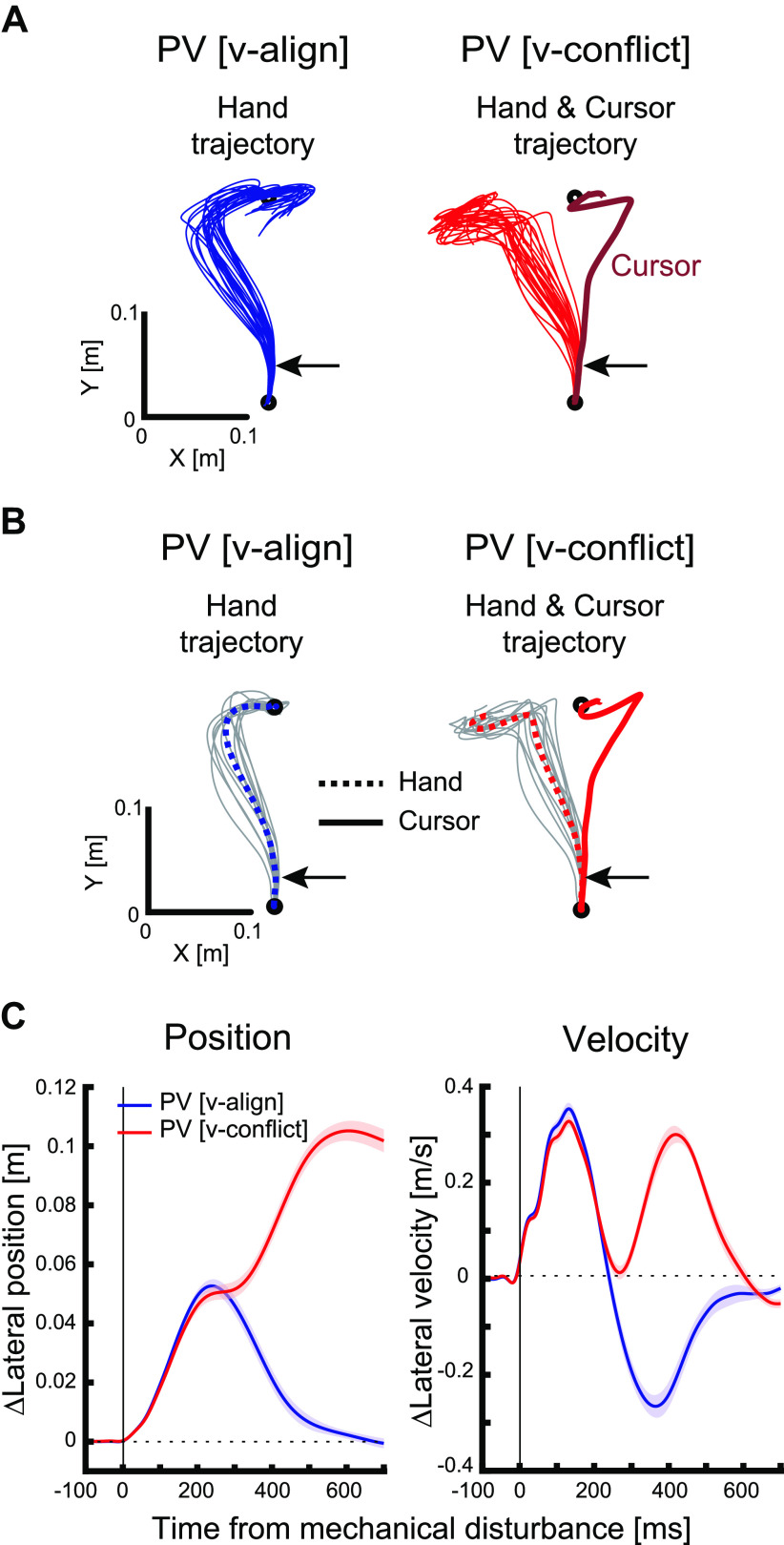
Kinematics during *experiment 2*. *A*: trajectory data from an exemplar participant. Hand trajectories (each trial) in the trial type of mechanical disturbance with aligned visual feedback (PV [v-align]; *left*), and hand (each trial) and cursor (across-trials average) trajectories in the trial type of mechanical and visual disturbance with conflicting visual feedback (PV [v-conflict]; *right*). Black arrows indicate the timing of mechanical loads. *B*: across-participants average trajectories. Gray lines indicate across-trials average of each participant, dotted colored lines indicate across-participants average of hand trajectories, and the solid colored line in the *right* panel indicates across-participants average of cursor trajectories. *C*: position and velocity changes. Each line indicates across-participants average in each trial type and shaded areas indicate mean ±1 SE. Average of baseline trials was subtracted. Blue lines indicate the trial type of mechanical disturbance with aligned visual feedback, and red lines indicate mechanical and visual disturbance with conflicting visual feedback. Data were aligned on the mechanical disturbance onset.

Human participants performed similarly in *experiment 2* when visual feedback was aligned or in conflict with proprioceptive feedback (Fig. 6). Figure 6*A* illustrates hand and cursor trajectories for a single subject when the proprioceptive and visual feedback of the arm were aligned (PV [v-align]) as compared with when they were in opposite directions (PV [v-conflict]). Mean trajectories for each subject are displayed in Fig. 6*B*. In both trial types, the hand was bumped away by a mechanical load (black arrows in the figure) soon after the participant left the start target. Trials in which feedback was aligned with the mechanical disturbance led to a corrective response to return to the spatial goal with peak lateral deviation occurring at ∼250 ms postdisturbance. Lateral hand motion for trials in which visual feedback was in the opposite direction were initially similar up to peak lateral deviation, but then the hand moved more laterally to return the cursor toward the spatial goal. Differences in lateral hand position across feedback conditions (aligned vs. conflict) were first observed at 271 ms (extension) and 248 ms (flexion), and differences in lateral hand velocity was first observed at 210 ms (extension) and 191 ms (flexion) after the mechanical disturbances were applied (Fig. 6*C*).

**Figure 7.**
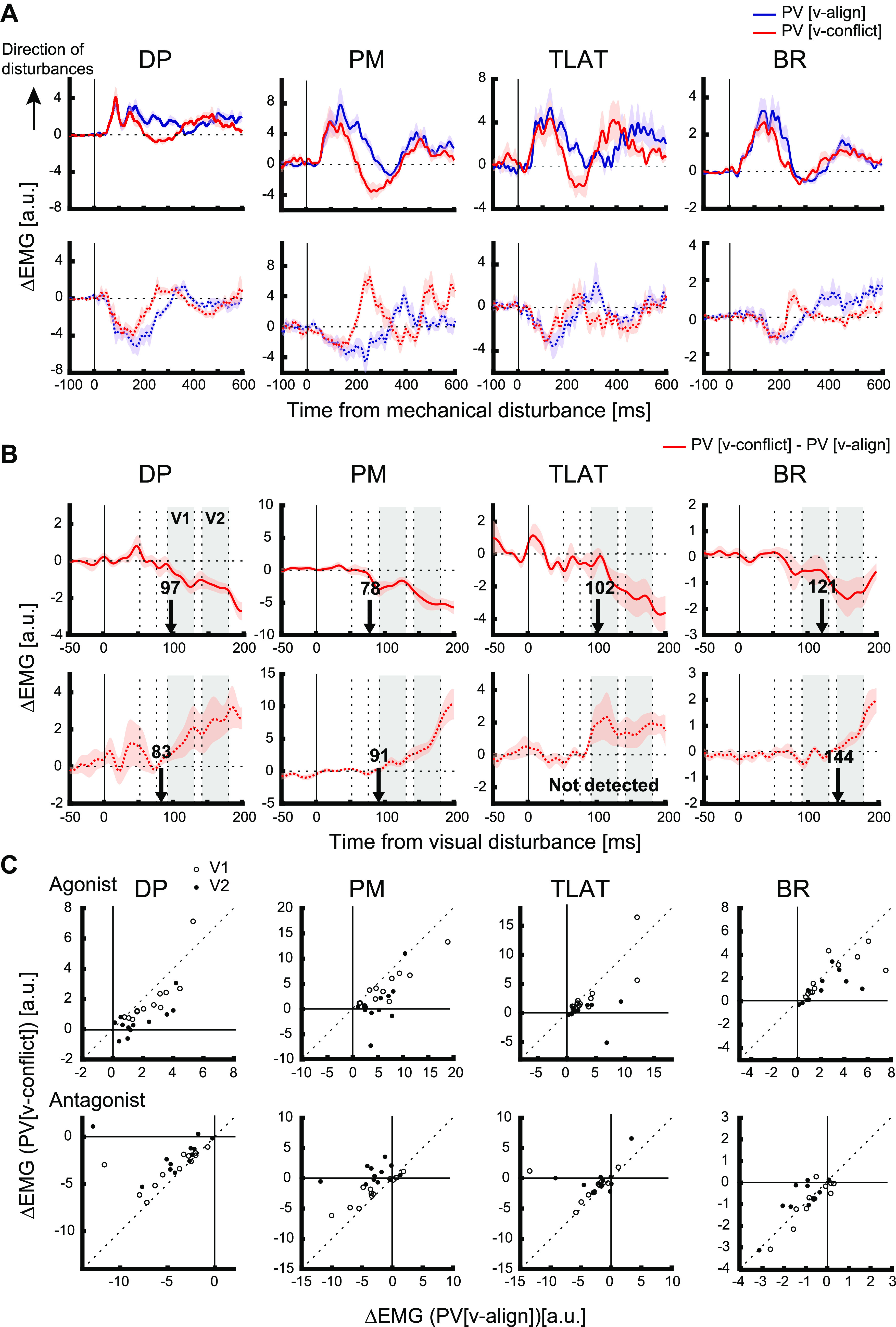
Muscle responses during *experiment 2*. *A*: across-participants average of muscle activities. Blue lines indicate the trial type of mechanical disturbance with aligned visual feedback, and red lines indicate of mechanical and visual disturbance with conflicting visual feedback. Data were aligned on the mechanical disturbance onset. Shaded areas indicate mean ± 1 SE. *B*: across-participants average muscle response of the trials of mechanical and visual disturbance with conflicting visual feedback in which the mean muscle response for the mechanical disturbance with aligned visual feedback was subtracted (“PV [v-conflict] − PV[v-align]”). The gray boxes in each panel indicate V1 (90–130 ms) and V2 (140–180 ms) epochs. Data were aligned on the visualization onset. Black arrows indicate the timepoint in which differences in the muscle response across the mechanical and visual disturbance with conflicting visual feedback and the mechanical disturbance with aligned visual feedback were detected. *C*: scatter plots of muscle responses of the trials of mechanical and visual disturbance with aligned visual feedback (“PV[v-align]”) and that with conflicting visual feedback (“PV [v-conflict]”). Each dot indicates each participant, and open circles indicate V1 and filled circles indicate V2 epochs. The *top* row shows responses for agonist disturbance and the *bottom* row shows responses for antagonist disturbance. a.u., Arbitrary units; BR, brachioradialis; DP, posterior deltoid; EMG, electromyography; PM, pectoralis major; PV, mechanical and visual disturbance; [v-align], visual feedback present and aligned with hand; [v-conflict], visual disturbance opposite direction to mechanical disturbance; TLAT, lateral head of the triceps.

As expected, muscle responses were initially similar whether visual feedback was aligned or in the opposite direction to the mechanical disturbance. Figure 7*A* displays EMG responses for all participants, respectively, with the data aligned on the onset of the mechanical disturbance. The mean muscle response when visual and mechanical disturbances were aligned was subtracted from each trial in which visual feedback was in conflict. Figure 7*B* displays these differences after the trials were realigned to the visual disturbance. The mean EMG traces for all muscles showed similar deviations from zero. It shows that when visual feedback was in conflict, muscle responses decreased around V1 epoch, indicating that the ongoing response to mechanical disturbance was reduced. Similar trends were observed when looking at individual participants as shown in the scatter plots comparing PV [v-conflict] and PV [v-align] in Fig. 7*C*. For the agonist direction, most points lie below the unity line, whereas for the antagonist direction, they lie above the unity line. These results indicate that the change in muscle activity for PV [v-conflict] was smaller than for PV [v-align].

To quantify the difference in the EMG response between trial types (i.e., PV [v-align] and PV [v-conflict]) for V1 and V2 epochs, we performed a two-way repeated-measures ANOVA (2 trial types × 2 epochs). For agonist disturbances, the main effect was significant for trial type in all muscles (DP, *F*_1,11_ = 26.21, *P* < 0.001; PM, *F*_1,11_ = 58.91, *P* < 0.001; TLAT, *F*_1,11_ = 10.88, *P* = 0.007; BR, *F*_1,10_ = 21.99, *P* = 0.001). The main effect of epoch was also significant in all muscles (DP, *F*_1,11_ = 43.48, *P* < 0.001; PM, *F*_1,11 _= 32.04, *P* < 0.001; TLAT, *F*_1,11 _= 18.51, *P* = 0.001; BR, *F*_1,10 _= 12.37, *P* = 0.006). The interaction effect (trial type × epoch) was not significant in all muscles (DP, *P* = 0.118; PM, *P* = 0.060; TLAT, *P* = 0.109; BR, *P* = 0.149). A post hoc test indicate a significant difference between PV [v-align] and PV [v-conflict] in V1 (90–130 ms) only for PM, whereas it was significant in V2 (140–180ms) for all muscles (DP, V1 [*P* = 0.204], V2 [*P* = 0.003]; PM, V1 [*P* = 0.014], V2 [*P* < 0.001]; TLAT, V1 [*P* = 1.000], V2 [*P* = 0.027]; BR, V1 [*P* = 0.309], V2 [*P* = 0.008], Bonferroni corrected). For antagonist disturbances, a significant main effect of trial type was found in all except for BR (DP, *F*_1,11_ = 72.37, *P* < 0.001; PM, *F*_1,11_ = 60.99, *P* < 0.001; TLAT, *F*_1,11 _= 28.83, *P* < 0.001; BR, *F*_1,10_ = 0.52, *P* = 0.488), and the main effect of epoch was significant in all except for BR as well (DP, *F*_1,11_ = 17.13, *P* = 0.002; PM, *F*_1,11 _= 31.11, *P* < 0.001; TLAT, *F*_1,11 _= 14.83, *P* = 0.003; BR, *F*_1,10_ = 3.23, *P* = 0.102). The interaction effect was significant in all except for TLAT (DP, *P* = 0.043; PM, *P* = 0.001; TLAT, *P* = 0.648; BR, *P* = 0.009). A post hoc test indicated a significant difference between PV [v-align] and PV [v-conflict] in V1 in DP and TLAT, and in V2, in all except for BR (DP, V1 [*P* = 0.006], V2 [*P* < 0.001]; PM, V1 [*P* = 0.197], V2 [*P* < 0.001]; TLAT, V1 [*P* = 0.010], V2 [*P* = 0.032]; BR, V1 [*P* = 0.637], V2 [*P* = 0.114]).

Similar to *experiment 1*, there were a number of outliers that could impact our ANOVA results, and thus we reran our analysis using a robust ANOVA. For agonist disturbances, the main effect of trial type was significant for each muscle except BR (DP, *F*_1,12_ = 5.86, *P* = 0.03; PM, *F*_1,12_ = 10.3, *P* = 0.004; TLAT, *F*_1,12_ = 6.3, *P* = 0.02; BR, *F*_1,11 _= 1.1, *P* = 0.3). Post hoc indicated a significant difference between PV [v-align] and PV [v-conflict] in V2 for DP, PM, and TLAT (DP, *P* = 0.01; PM, *P* = 0.008; TLAT, *P* = 0.04).

For antagonist disturbances, significant main effect of trial type was found in all except for TLAT and BR (DP, *F*_1,12 _= 4.76, *P* = 0.04; PM, *F*_1,12 _= 6.3, *P* = 0.02; TLAT, *F*_1,12_ = 3.4, *P* = 0.08; BR, *F*_1,11_ = 0.19, *P* = 0.67). Post hoc indicated a significant difference between PV [v-align] and PV [v-conflict] in V2 for DP and PM (DP, *P* = 0.04; PM, *P* = 0.001; BR, *P* = 0.8).

Similar to *experiment 1*, we also investigated if the muscle response changed across trials by comparing the first and last five trials of each type of disturbance. We show the results for agonist disturbances and mechanical disturbance with conflicting visual feedback (PV[v-conflict]) here only. For visual disturbance (V [v-shift]), a significant main effect of the trial bin (i.e., first/last 5 trials) was detected in a two-way repeated-measures ANOVA (2 trial bins × 2 epochs) in DP and TLAT (DP, *F*_1,11 _= 11.80, *P* = 0.006; TLAT, *F*_1,11_ = 21.99, *P* = 0.001). A post hoc Bonferroni test indicated a significant decrease of muscle responses in V1 for DP ([*P* = 0.028]) and significant increase in TLAT ([*P* = 0.009]) on the other hand.

We also examined the earliest timepoint in which there was a difference in the muscle response across the two trial types using ROC analysis (black arrows in Fig. 7*B*). The beginning of the change in the muscle response can be estimated by using a regression to the “knee,” the point of inflexion in the ROC based on crossing the 0.75 threshold (22, 24). Median times for a difference in the EMG response based on the knee was 100 [interquartile range of subjects, 83–116] ms for agonist disturbances and 91 [87–118] ms for antagonist disturbances. Note that the response time was not detected in our criteria in the antagonist disturbance for TLAT. Thus, visually mediated motor responses remain potent even during the presence of a mechanical disturbance. In that regard, we can assert that the results of *experiment 1* indeed reflected an integration rule, which we showed was compatible with dynamic Bayesian estimation.

## DISCUSSIONS

In the current study, we addressed how visual and proprioceptive feedback are integrated together to generate motor corrections. We show that muscle responses to mechanical disturbances were not substantively altered by visual feedback of the limb. However, visual feedback could strongly influence muscle responses when there was a conflict between the visual goal and proprioceptive feedback of limb motion.

A recent review proposed a model for sensory integration across multiple feedback pathways (19). They hypothesized that in the earliest stage of control, each feedback module estimates the state of the body independently. This enables the motor system to quickly correct ongoing movement under an unpredictable environment. Subsequently, a later stage of control contributes to maximize reliability of state estimates, by integrating information from multiple sensory modalities.

Supporting this framework, previous studies highlight that there may be two visual feedback pathways, a transcortical pathway involving parietal and motor cortices (35–37) and a subcortical pathway through superior colliculus (15–17, 24, 38–41). Recently, we have provided behavioral evidence for a two- stage visual feedback process (10). Specifically, we observed that the earliest visual response from 90 to 130 ms did not consider the presence of obstacles which only influenced muscle responses starting at 130 ms.

This two-stage pattern of feedback processing is also present for proprioceptive feedback, with the earliest feedback response beginning 25 ms after a load reflecting a spinal process that considers a few factors such the magnitude of the load (27, 42, 43, but see Ref. 44). Subsequently, a second cortical feedback response beginning at 60 ms considers a multitude of goal-related factors such as task redundancy, obstacles, and urgency (6, 30, 45–47). Collectively, these results support the idea of a two-stage process for both proprioceptive and visual feedback. Initially, there are separate subcortical processes (spinal cord and superior colliculus, respectively) occurring first followed by a later cortical process with proprioceptive and visual feedback combined which can influence muscle responses starting 60 ms after a mechanical disturbance and 130 ms after a visual disturbance.

One observation from *experiment 1* was that muscle responses to proprioceptive and visual feedback are subadditive in the 140–180 ms epoch. Indeed, the difference between the muscle responses for mechanical loads with and without vision (i.e., PV[v-align]-P[no-v]) was considerably smaller than for the cursor-only perturbation (V[v-shift]). This provides strong evidence that during a time period when feedback may involve a common pathway that the resulting motor response reflects a subadditive combination of the proprioceptive and visual feedback responses, supporting our main hypothesis.

Thus after 140 ms, our results are more aligned with the predictions from a dynamic Bayesian model, where sensory signals are integrated using an internal model that accounts for sensory delays (18). Simulations using a dynamic Bayesian model predict that qualitatively similar feedback corrections to mechanical disturbances are observed whether visual feedback is present or not. Critically, this model predicts virtually no change in the muscle response whether visual feedback is present or absent, and predicts a reduction in end point errors at the end of movement when visual feedback is provided. The results from *experiment 1* provide empirical support to these model features. The model suggests that this behavior arises due to an extrapolation of sensory signals, which requires knowledge of the delay and internal models of limb dynamics during this time interval (48–50). Specifically, the motor response was subadditive in the model as the state estimate had already been updated based on the error from proprioceptive feedback before visual feedback had arrived. As a result, the difference between visual feedback and the state estimate was already close to zero, causing little change in the control output.

However, it is less clear whether muscle responses were subadditive in the early 90–130 ms epoch as we found subadditive muscle responses in only two out of eight muscle samples. One challenge is that the size of the muscle response to the cursor shift is relatively small in this epoch. Note that this difference was also small for the model. Thus, it is possible that during this epoch, muscle responses reflect either an integration or the output of separate pathways for vision and proprioception. Future studies are required to determine whether visual response are subadditive in this early epoch.

Unlike our previous model (18), the current model assumed that vision and proprioception updated separate position state variables and that the goal was to get the cursor to the target. This was needed so that the controller moved the cursor position to the goal instead of a weighted average of the hand and cursor positions, thus matching human performance in this task. However, having separate cursor and hand positions appears to conflict with several studies indicating that the motor system combines proprioception and vision together when estimating hand position (51, 52). This difference could be attributed to experimental design as those studies only provided visual feedback during the planning phase of the movement and the participants were unaware of experimenter-imposed visual errors. In contrast, in our study, visual feedback was provided throughout the movement and the participants had to get the cursor to the goal to complete the task successfully. Also, we believe participants were aware of the errors introduced by the cursor perturbations, although we did not ask them explicitly. It is worth noting as well that our main finding from the model would be unaffected had we integrated the cursor and hand positions into the same state variable as subadditive muscle responses would still have emerged (data not shown). Thus, the motor system may be able to switch between these strategies of either keeping visual and proprioceptive feedback about position separate (as in our study) or integrating them together (51, 52) that is dependent on the task structure or perturbation size.

In the present study, we used gradual shifts of the cursor that mirrored the trajectory generated when a mechanical disturbance is applied to directly compare the size of the feedback response across sensory modalities. Our model predicted that the muscle response for the visual disturbance would be delayed (reflecting the imposed sensory delay in the model) and smaller than the muscle response for the mechanical load. There are several factors for why muscle responses for mechanical loads were larger than for visual responses in the model. First, mechanical loads impact state estimates of limb motion (i.e., position and velocity) and applied loads, which were directly observable by proprioceptive feedback (Fig. 2*A*). In contrast, visual disturbances only impacted state estimates of limb motion, and vision only provided feedback about the errors in the position and velocity of the cursor to the model. Second, for visual disturbances, there is a conflict between visual and proprioceptive feedback. Although in our model the cursor and hand positions are separate state variables, we assume vision and proprioception observe the same velocity state variable. Thus, following a visual disturbance, vision indicates an error in the velocity of the cursor, whereas there is no error signaled by proprioceptive feedback. In contrast, for the mechanical-only disturbance, vision is removed and thus there is no conflict between proprioceptive and visual feedback. Indeed, accounting for these two factors in the model can result in motor output for visual disturbances that are comparable in magnitude with the mechanical disturbances (data not shown). Importantly, we found human muscle responses were larger for mechanical disturbances than visual disturbances, which may reflect either limited observability of the state vector by visual feedback or a conflict between proprioception and vision, or a combination of the two factors.

One alternate explanation of our results may be a credit assignment mechanisms that the motor system corrects the errors based on the belief of where the error is arising from (53–55). However, we do not think such a mechanism would influence our results. Our previous study identified that motor responses scale with the magnitude of a cursor jump up to 2 cm where the motor response started to plateau or even decrease for jumps of 8 cm (10). This reduction for large cursor jumps likely reflects the fact that it is impossible for the hand to shift 8 cm instantly and thus leads to a reduced response as it not clear that the appearance of a shifted cursor 8 cm from its previous position plausibly reflects movement of the hand in space. Critically, although our cursor motion was deviated by ∼4 cm, we used gradual shifts of the cursor making them far more biologically plausible. We also assume that the relationship between credit assignment and dynamic Bayesian integration mechanisms are hierarchical and think there are interesting issues on how these two levels of processing may interact which deserves further study.

A limitation in the present study was that the temporal pattern of the visual-only disturbance was greater than the trajectory of the hand during the mechanical disturbance. The lateral motion of the cursor shift for the visual disturbance was matched to the motion of the hand for mechanical disturbance from a calibration session collected before the main experiment session. These differences suggest that the motor response may have changed over time. We also found a decrease in motor responses across trials in the main experiment in both trials with visual disturbances and those with mechanical disturbances for several muscles. A recent study (56) suggested that such a decrease in sensitivity to perturbations as compensation for disturbances over fast time scales may reflect a robust control strategy that enables humans and animals to quickly handle unpredictable changes in disturbances without adaptation. However, scaling down the muscle responses for the visual disturbance trials (Fig. 4*B*) showed that the larger magnitude of the visual disturbance in the early phase of movements did not impact our main focus on how these visual and proprioceptive feedback are combined together. Future studies that attempt to match different types of motor responses may need to develop a protocol in which disturbance magnitude or temporal pattern is constantly altered based on the present performance of the participant.

The present results highlight that visual and proprioceptive feedback are combined in a complex manner during goal-directed motor actions. Vision contributes minimally to correct motor actions during an ongoing mechanical disturbance but is quite potent when there is a conflict between visual and proprioceptive feedback. This conflict between sensory modalities was possible in the present and in many previous studies because a cursor was used to represent hand position (10, 13, 57, 58). In effect, the cursor is a tool, and from this perspective, the goal of the task is to move the visual tool to the visually defined spatial goal. As a result, visual feedback is essential once the cursor and hand become misaligned. Physical tools provide a less extreme but more interesting problem when integrating vision with somatosensory feedback, as muscle and cutaneous information can be used to estimate tool motion, but the accuracy of these estimates depend on many factors, such as size of the tool, the physical properties of the tool (i.e., rigid vs. flexible), and of course, the skill of the individual to use that tool. Inevitably, this will start to shift the importance of visual feedback from that observed when simply reaching to a spatial goal that we predict will continue to follow a dynamic Bayesian model.

## SUPPLEMENTAL DATA

https://github.com/KevinCross/DynamicBayesianModel Supplemental Table S1: https://github.com/KevinCross/DynamicBayesianModel.

10.5281/zenodo.5725741 Supplemental Fig. S1: https://doi.org/10.5281/zenodo.5725741.

## GRANTS

This work was supported by National Sciences and Engineering Research Council (NSERC) (to S.H.S.) and Japanese Society for the Promotion of Science Overseas Research Fellowships (to S.K.).

## DISCLOSURES

S.H.S. is associated with Kinarm, which commercializes the robotic device used in present study. None of the other authors has any conflicts of interest, financial or otherwise, to disclose. 

## AUTHOR CONTRIBUTIONS

S.K. and S.H.S. conceived and designed research; S.K. performed experiments; S.K. and P.B. analyzed data; S.K., F.C., K.P.C., P.B., and S.H.S. interpreted results of experiments; S.K., F.C., K.P.C., P.B., and S.H.S. prepared figures; S.K. and S.H.S. drafted manuscript; S.K., F.C., K.P.C., P.B., and S.H.S. edited and revised manuscript; S.K., F.C., K.P.C., P.B., and S.H.S. approved final version of manuscript,

## ENDNOTE

At the request of the authors, readers are herein alerted to the fact that model code and data are available at https://github.com/KevinCross/DynamicBayesianModel. These materials are not a part of this manuscript and have not undergone peer review by the American Physiological Society (APS). APS and the journal editors take no responsibility for these materials, for the website address, or for any links to or from it.
